# Current-induced forces in mesoscopic systems: A scattering-matrix approach

**DOI:** 10.3762/bjnano.3.15

**Published:** 2012-02-20

**Authors:** Niels Bode, Silvia Viola Kusminskiy, Reinhold Egger, Felix von Oppen

**Affiliations:** 1 Dahlem Center for Complex Quantum Systems and Fachbereich Physik, Freie Universität Berlin, 14195 Berlin, Germany; 2 Institut für Theoretische Physik, Heinrich-Heine-Universität, D-40225 Düsseldorf, Germany

**Keywords:** current-induced forces, electronic transport theory, nanoelectromechanical systems, scattering matrix, S-matrix

## Abstract

Nanoelectromechanical systems are characterized by an intimate connection between electronic and mechanical degrees of freedom. Due to the nanoscopic scale, current flowing through the system noticeably impacts upons the vibrational dynamics of the device, complementing the effect of the vibrational modes on the electronic dynamics. We employ the scattering-matrix approach to quantum transport in order to develop a unified theory of nanoelectromechanical systems out of equilibrium. For a slow mechanical mode the current can be obtained from the Landauer–Büttiker formula in the strictly adiabatic limit. The leading correction to the adiabatic limit reduces to Brouwer’s formula for the current of a quantum pump in the absence of a bias voltage. The principal results of the present paper are the scattering-matrix expressions for the current-induced forces acting on the mechanical degrees of freedom. These forces control the Langevin dynamics of the mechanical modes. Specifically, we derive expressions for the (typically nonconservative) mean force, for the (possibly negative) damping force, an effective “Lorentz” force that exists even for time-reversal-invariant systems, and the fluctuating Langevin force originating from Nyquist and shot noise of the current flow. We apply our general formalism to several simple models that illustrate the peculiar nature of the current-induced forces. Specifically, we find that in out-of-equilibrium situations the current-induced forces can destabilize the mechanical vibrations and cause limit-cycle dynamics.

## Introduction

Scattering theory has proved to be a highly successful method for treating coherent transport in mesoscopic systems [1]. Part of its appeal is rooted in its conceptual simplicity: Transport through a mesoscopic object can be described in terms of the transmission and reflection of electronic waves that are scattered by a potential. This approach was introduced by Landauer [2–3] and generalized by Büttiker et al. [4] and leads to their well-known formula for the conductance of multiterminal mesoscopic conductors. For time-dependent phenomena, scattering-matrix expressions have been obtained for quantum pumping [5–6], a process by which a direct current is generated through temporal variations of relevant parameters of the system, such as a gate voltage or a magnetic field. The case of pumping in an out-of-equilibrium, biased system has remained largely unexplored so far [7–8].

The purpose of the present paper is to further develop the scattering-matrix approach into a simple, unifying formalism to treat nanoelectromechanical systems (NEMS). The coupling between mechanical and electronic degrees of freedom is the defining characteristic of NEMS [9–10], such as suspended quantum dots [11], carbon nanotubes or graphene sheets [12–13], one-dimensional wires [14], and molecular junctions [15–16]. For these systems, a transport current can excite mechanical modes, and vice versa, the mechanical motion affects the transport current. The reduced size and high sensitivity of the resulting devices make them attractive for applications such as sensors of mass or charge, nanoscale motors, or switches [17]. On a more fundamental level, the capability of cooling the system by means of back-action allows one to study quantum phenomena at the mesoscopic level, eventually reaching the quantum limit of measurement [18–19].

All of these applications require an understanding of the mechanical forces that act on the nanoelectromechanical system in the presence of a transport current. These are referred to as *current-induced forces*, and have been observed in seminal experiments [20–21]. Recently we have shown that it is possible to fully express the current-induced forces in terms of a scattering matrix formalism, for arbitrary (albeit adiabatic) out-of-equilibrium situations [22], thus providing the tools for a systematic approach to study the interplay between electronic and mechanical degrees of freedom in NEMS.

In the context of NEMS, two well-defined limits can be identified at which electronic and mechanical time scales decouple, and which give rise to different experimental phenomena. On one side, when the electronic time scales are slow compared with the mechanical vibrations, drastic consequences can be observed for the electronic transport, such as side bands due to phonon-assisted tunneling [23–24] or the Frank–Condon blockade effect, a phononic analogue of the Coulomb blockade in quantum dots [25–27]. In the opposite regime, electrons tunnel through the nanostructure rapidly, observing a quasistatic configuration of the vibrational modes, but affecting their dynamics profoundly at the same time [18–21]. It is on this regime that our present work focuses. We treat the vibrational degrees of freedom as classical entities embedded in an electronic environment: Pictorially, many electrons pass through the nanostructure during one vibrational period, impinging randomly on the modes. In this limit, it is natural to assume that the dynamics of the vibrational modes, represented by collective coordinates *X*_ν_, will be governed by a set of coupled Langevin equations

[1]
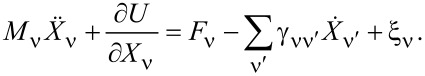


Here we have grouped the purely elastic contribution on the left-hand side (LHS) of Equation 1, *M*_ν_ being the effective mass of mode ν and *U*(**X**) an elastic potential. On the right-hand side (RHS) we collected the current-induced forces: The mean force *F*_ν_, a term proportional to the velocity of the modes 
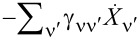
, and the Langevin fluctuating forces ξ_ν_. The main results of our work are expressions for the current-induced forces in terms of the scattering matrix and its parametric derivatives. These are given by Equation 2 for the mean force *F*_ν_(**X**), Equation 3 for the correlator *D*_νν′_(**X**) of the stochastic force ξ_ν_, and Equation 4, and Equation 5 for the two kinds of forces (dissipative-friction force and effective “Lorentz” force, as we discuss below) encoded by the matrix γ_νν′_(**X**).

These forces have been previously studied theoretically within different formalisms. The case of one electronic level coupled to one vibrational mode was studied within a Green’s function approach in [28–29], in which the authors showed that the current-induced forces can lead to a bistable effective potential and consequently to switching. In [30], the authors studied the case of multiple vibrational modes within a linear approximation, finding a Lorentz-like current-induced force arising from the electronic Berry phase [31]. In simple situations, the current-induced forces have been also studied within a scattering matrix approach in the context of quantum measurement back-action [32] (see also [33]), momentum-transfer statistics [34], and of magnetic systems to describe Gilbert damping [35]. Current-induced forces have been shown to be of relevance near mechanical instabilities [36–38] and to drive NEMS into instabilities and strong nonlinear behavior [39–41]. Our formalism allows us to retain the nonlinearities of the problem, which is essential for even a qualitative description of the dynamics, while turning the problem of calculating the current-induced forces into a scattering problem for which standard techniques can be applied.

In what follows, we develop these ideas in detail, giving a thorough derivation of the expressions in terms of the scattering matrix for the current-induced forces found in [22], and include several applications to specific systems. Moreover, we extend the theoretical results of [22] in two ways. We treat a general coupling between the collective modes *X*_ν_ and the electrons, generalizing the linear coupling expressions obtained previously. We also allow for an arbitrary energy dependence in the hybridization between the leads and the quantum dot, allowing more flexibility for modeling real systems. In the section “Microscopic derivation of the Langevin equation”, we introduce the theoretical model, and derive the equations of motion of the mechanical degrees of freedom starting from a microscopic Hamiltonian. We show how the Langevin equation, Equation 1, emerges naturally from a microscopic model when employing the nonequilibrium Born–Oppenheimer (NEBO) approximation, appropriate for the limit of slow vibrational dynamics, and derive the current-induced forces in terms of the microscopic parameters. In the section “S-matrix theory of current-induced forces”, we show that the current-induced forces can be written in terms of parametric derivatives of the scattering matrix (S-matrix) of the system, and state general properties that can be derived from S-matrix symmetry considerations. In the section “Current”, we complete the discussion of nanoelectromechanical systems in terms of scattering matrices by providing a corresponding expression for the charge current. In the section “Applications”, we apply our formalism to simple models of increasing complexity, namely a single resonant level, a two-level model, and a two-level/two-mode model. For better readability, we have relegated part of some lengthy calculations to Supporting Information File 1, together with a list of useful relations that are used throughout the main text.

## Results and Discussion

### Microscopic derivation of the Langevin equation

#### The model

We model the system as a mesoscopic quantum dot connected to multiple leads and coupled to vibrational degrees of freedom. Throughout this work we consider noninteracting electrons and we set 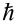 = 1. The Hamiltonian for the full system reads

[6]
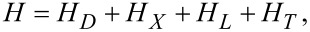


where the different terms are introduced in the following.

We describe the quantum dot by *M* electronic levels coupled to *N* slow collective degrees of freedom 
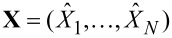
. This is contained in the Hamiltonian of the dot

[7]
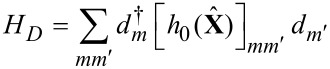


which describes the electronic levels of the dot and their dependence on the coordinates of the collective modes, 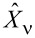 (
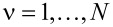
), by the hermitian *M* × *M* matrix *h*_0_(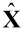). The operator *d*^†^ (*d*) creates (annihilates) an electron in the dot and the indices *m, m*′ (= 1,…,*M*) label the electronic levels. Note that here we generalize our previous results obtained for a linear coupling in 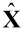 [22], and allow *h*_0_ to be a general function of 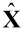. Our analysis is valid for any coupling strength. The free evolution of the “mechanical” degrees of freedom of the dot is described by the Hamiltonian

[8]
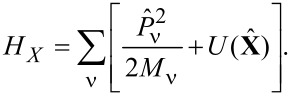


The leads act as electronic reservoirs kept at fixed chemical potentials μ_α_ and are described by

[9]
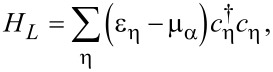


where we represent the electrons in the leads by the creation (annihilation) operators *c*^†^ (*c*). The electrons in the leads obey the Fermi–Dirac distribution


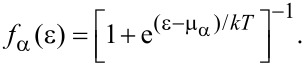


The leads are labeled by α = 1,…,*L*, each containing channels *n* = 1,…,*N*_α_. We combine η = (α,*n*) into a general “lead” index, 
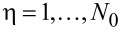
 with 
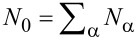
.

Finally, the Hamiltonian *H_T_* represents the tunneling between the leads and the levels in the dot,

[10]
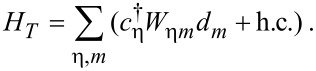


#### Nonequilibrium Born–Oppenheimer approximation

We use as a starting point the Heisenberg equations of motion for the mechanical modes, which can be cast as

[11]
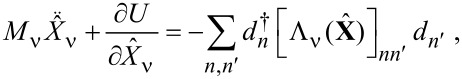


where we have introduced the 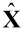-dependent matrices

[12]
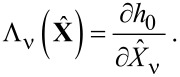


The RHS of Equation 11 contains the current-induced forces, expressed through the electronic operators *d* of the quantum dot. We now proceed to calculate these forces within a nonequilibrium Born–Oppenheimer (NEBO) approximation, in which the dynamics of the collective modes is assumed to be slow. In this limit, we can treat the mechanical degrees of freedom as being classical, acting as a slow classical field on the fast electronic dynamics.

The NEBO approximation involves averaging the RHS of Equation 11 over times long compared to the electronic time scale, but short in terms of the oscillator dynamics. In this approximation, the force operator is represented by its (average) expectation value 
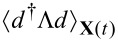
, evaluated for a given trajectory **X**(*t*) of the mechanical degrees of freedom, plus fluctuations containing both Johnson–Nyquist and shot noise. These fluctuations give rise to a Langevin force ξ_ν_. Hence Equation 11 becomes

[13]
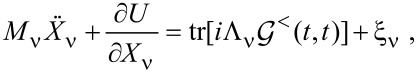


where the trace “tr” is taken over the dot levels, and we have introduced the lesser Green’s function

[14]
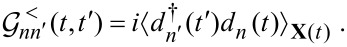


The variance of the stochastic force ξ_ν_ is governed by the symmetrized fluctuations of the operator *d*^†^Λ*d*. Given that the electronic fluctuations happen on short time scales, ξ_ν_ is locally correlated in time,

[15]
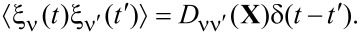


(An alternative, but equivalent, derivation is based on a saddle-point approximation for the Keldysh action, see, e.g., [42]). Since we are dealing with noninteracting electrons, *D*(**X**) can be expressed in terms of single particle Green’s functions by using Wick’s theorem. This readily yields

[16]
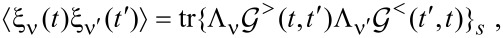


where

[17]
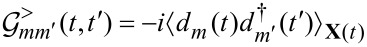


is the greater Green’s function. These expressions for the current-induced forces show that we need to evaluate the electronic Green’s function for a given classical trajectory **X**(*t*). In doing so, we can exploit the assumption that the mechanical degrees of freedom are slow compared to the electrons. Thus, we can approximate the Green’s function by its solution to first order in the velocities 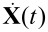. We now proceed with this derivation, starting with the Dyson equation for the retarded Green’s function

[18]
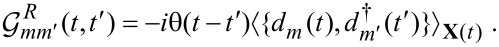


Here {.,.} indicates the anticommutator. We note that since we consider noninteracting electrons, we can restore the lesser and greater Green’s functions (or the advanced Green’s function 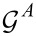) at the end of the calculation by standard manipulations.

The hybridization with the leads is taken into account through the self-energy [43]

[19]
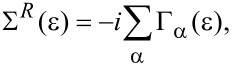


which is given in terms of the width functions

[20]
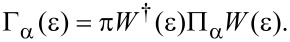


Here we have defined Π_α_ as a projection operator onto lead α and absorbed the square root factors of the density of states in the leads into the coupling matrix *W* for notational simplicity. Note that we allow *W* to depend on energy. (Compare with the wide-band limit discussed in [22], which employs an energy-independent hybridization Γ.)

Dyson’s equation for the retarded Green’s function can then be written, in matrix form, as

[21]
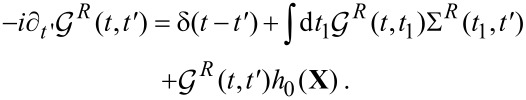


To perform the adiabatic expansion, it is convenient to work in the Wigner representation, in which fast and slow time scales are easily identifiable. The Wigner transform of a function *A*(*t*_1_,*t*_2_) depending on two time arguments is given by

[22]
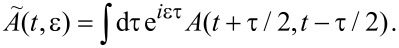


Using this prescription for the Green’s function 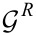, the slow mechanical motion implies that 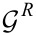 varies slowly with the central time *t* = (*t*_1_ + *t*_2_)/2 and oscillates fast with the relative time τ = *t*_1_ − *t*_2_. The Wigner transform of a convolution *C*(*t*_1_,*t*_2_) = ∫ d*t*_3_*A*(*t*_1_,*t*_3_)*B*(*t*_3_,*t*_2_) is given by

[23]
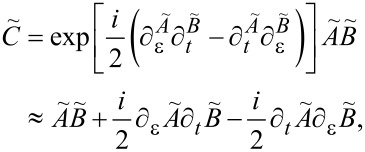


where we have dropped the higher-order derivatives in the last line, exploiting the slow variation with *t*. Therefore, using Equation 23 we can rewrite the Dyson equation (Equation 21) as

[24]
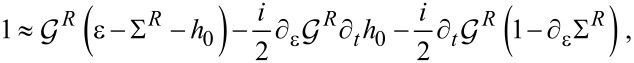


where the Green’s functions are now in the Wigner representation. Unless otherwise denoted by explicitly stating the variables, here and in the following all functions are in the Wigner representation. Finally, with the help of Equation 91 and Equation 92 from Supporting Information File 1, Section A, we obtain

[25]
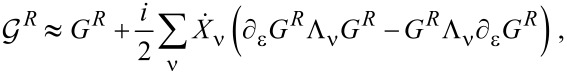


in terms of the strictly adiabatic Green’s function

[26]
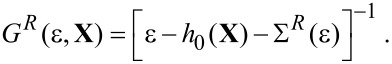


Our notation is such that 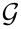 denotes *full* Green’s functions, while *G* denotes the strictly adiabatic (or *frozen*) Green’s functions that are evaluated for a fixed value of **X** (such that all derivatives with respect to central time in Equation 24 can be dropped). From now on, 
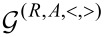
 denote the Green functions in the Wigner representation, with arguments (ε*,t*) and 
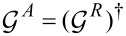
.

Using Langreth’s rule (see, e.g., [43])

[27]
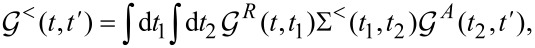


we can relate 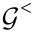 to 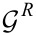. In Equation 27 we have introduced the lesser self energy ∑^<^, which in the Wigner representation takes the form

[28]
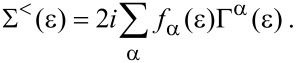


Note that ∑^<^ depends only on ε and is independent of the central time. Expanding Equation 27 up to the leading adiabatic correction according to Equation 23, we obtain 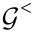 to first order in 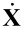,

[29]
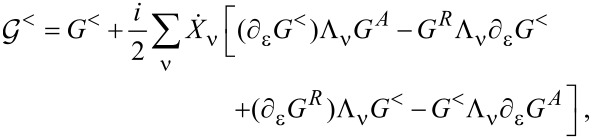


with *G*^<^ = *G^R^*∑^<^*G^A^*.

#### Current-induced forces in terms of Green’s functions

We can now collect the results from the previous section and identify the current-induced forces appearing in the Langevin Equation 1. Except for the stochastic noise force, the current-induced forces are encoded in 
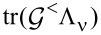
. In the strictly adiabatic limit, i.e., retaining only the first term on the RHS of Equation 29, 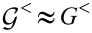, we obtain the mean force

[30]
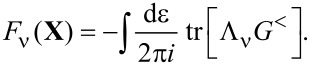


The leading-order correction in Equation 29 gives a velocity-dependent contribution to the current-induced forces, which determines the tensor γ_νν′_. After integration by parts, we find


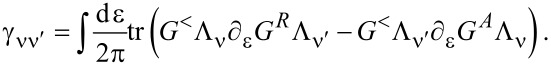


This tensor can be split into symmetric and antisymmetric contributions, γ = γ*^s^* + γ*^a^*, which define a dissipative term γ*^s^* and an orbital effective magnetic field γ*^a^* in the space of the collective modes. This interpretation is based on the fact that the corresponding force takes a Lorentz-like form. Using Equation 87 in Supporting Information File 1, Section A, and noting that 
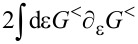
 = 
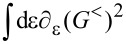
 = 0, we obtain the explicit expressions

[31]
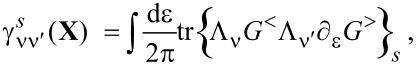


[32]
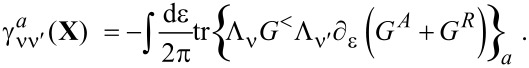


Here we have introduced the notation


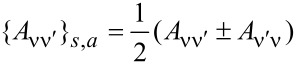


for symmetric and antisymmetric parts of an arbitrary matrix *A*.

Finally, the stochastic force ξ_ν_ is given by the thermal and nonequilibrium fluctuations of the force operator −*d*^†^Λ_ν_*d* in Equation 11. As indicated by the fluctuation–dissipation theorem, the fluctuating force is of the same order in the adiabatic expansion as the velocity-dependent force. Thus, we can evaluate the expression for the correlator *D*_νν′_(**X**) of the fluctuating force given in Equation 16 to lowest order in the adiabatic expansion, such that

[33]
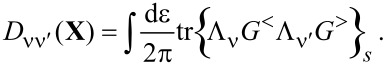


This formalism gives the tools needed to describe the dynamics of the vibrational modes in the presence of a bias for an arbitrary number of modes and dot levels. When Equation 30–Equation 32 are inserted back into Equation 1, they define a nonlinear Langevin equation due to their nontrivial dependencies on **X**(*t*) [28–29].

### S-matrix theory of current-induced forces

#### Adiabatic expansion of the S-matrix

Scattering matrix approaches to mesoscopic transport generally involve expressions in terms of the elastic S-matrix. For our problem, the S-matrix is elastic only in the strictly adiabatic limit, in which it is evaluated for a fixed value of **X**,

[34]
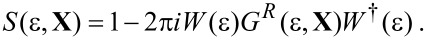


As pointed out by Moskalets and Büttiker [8,44], this is not sufficient for general out-of-equilibrium situations, even when **X**(*t*) varies in time adiabatically. In their work, they calculated, within a Floquet formalism, the leading correction to the strictly adiabatic S-matrix. We follow the same approach here, rephrased in terms of the Wigner representation. The full S-matrix can be written as [45] (note that, in line with the notation established before for the Green’s functions, the strictly adiabatic S-matrix is denoted by *S*, whereas the full S-matrix is denoted by 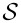)

[35]
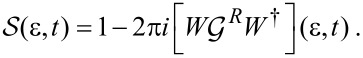


To go beyond the frozen approximation, we expand 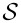 to leading order in 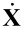,

[36]
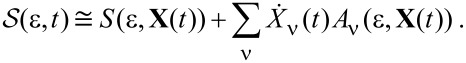


Thus, the leading correction defines the matrix *A*, which, similar to *S*, has definite symmetry properties. In particular, if the system is time-reversal invariant, the adiabatic S-matrix is even under time reversal, whereas *A* is odd. For a given problem, the A-matrix has to be obtained along with *S*.

We can now derive a Green’s function expression for the matrix *A* [46–47]. Comparing Equation 36 with the expansion to the same order of 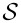 in terms of adiabatic Green’s functions (obtained in a straightforward manner by performing the convolution in Equation 35 explicitly and keeping terms up to 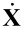) we obtain

[37]
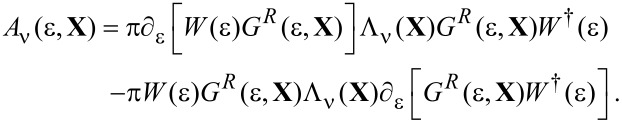


Current conservation constrains both the frozen and full scattering matrices to be unitary. From the unitarity of the frozen S-matrix, *S*^†^*S* = **1**, we obtain the useful relation

[38]
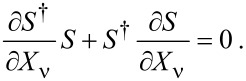


We will make use of Equation 38 repeatedly in the following sections. On the other hand, unitarity of the full S-matrix, 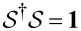, imposes a relation between the A-matrix and the frozen S-matrix. To first order in the velocity 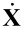 we have

[39]
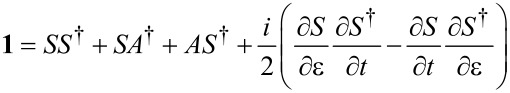


where *A*(ε,**X**) = ∑_ν_*A*_ν_(ε,**X**)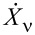. Therefore, *S* and *A* are related through

[40]
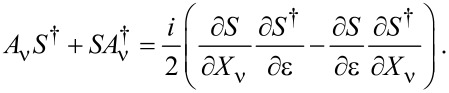


In the next section we will see that the A-matrix is essential to express the current-induced dissipation and “Lorentz” forces in Equation 31 and Equation 32.

#### Current-induced forces

**Mean force:** The mean force exerted by the electrons on the oscillator is given by Equation 30. Writing Equation 30 explicitly and using Equation 88 in Supporting Information File 1, Section A, we can express *G*^<^ in terms of *G^R^* and *G^A^* and obtain

[41]
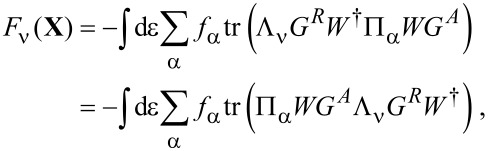


where the second equality exploits the cyclic invariance of the trace. Noting that, by Equation 93 in Supporting Information File 1, Section A,

[42]
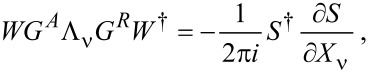


Equation 41 can be expressed directly in terms of scattering matrices *S*(ε,**X**) as

[2]
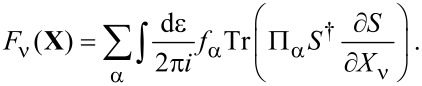


Note that now the trace (denoted by “Tr”) is over lead-space.

An important issue is whether this force is *conservative*, i.e., derivable from a potential. A necessary condition for this is a vanishing “curl” of the force,

[43]
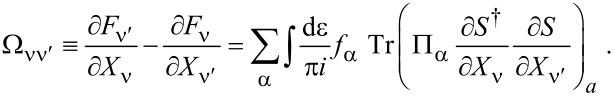


From Equation 43 it is seen that the mean force is conservative in thermal equilibrium, where Equation 43 can be turned into a trace over a commutator of finite-dimensional matrices: Indeed, in equilibrium the sum over the lead indices can be directly performed since *f*_α_ = *f* for all α, and ∑_α_Π_α_ = 1. Using the unitarity of the S-matrix and the cyclic property of the trace, we obtain:

[44]
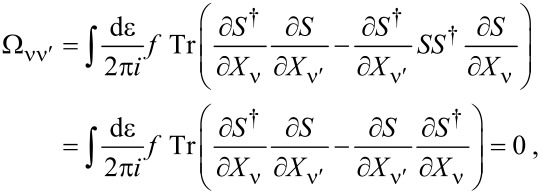


where in the last line we have used Equation 38. In general, however, the mean force will be *nonconservative* in out-of-equilibrium situations, providing a way to exert work on the mechanical degrees of freedom by controlling the external bias potential [30,48–49].

**Stochastic force:** Next, we discuss the fluctuating force ξ_ν_ with variance *D*_νν′_ given by Equation 33. Following a similar path as described in the previous subsection for the mean force *F*_ν_, we can also express the variance, Equation 33, of the fluctuating force in terms of the adiabatic S-matrix. Thus,

[3]
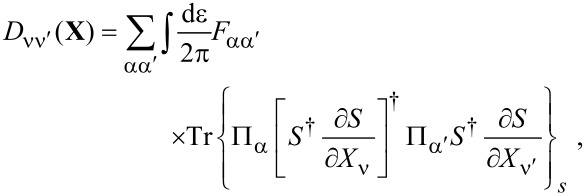


where we have introduced the function *F*_αα′_(ε) = *f*_α_(ε)[1 − *f*_α′_(ε)]. From Equation 3 it is straightforward to show that *D*_νν′_ is positive-definite. By performing a unitary transformation to a basis in which *D*_νν′_ is diagonal, using 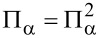 and the cyclic invariance of the trace, we obtain the expression

[45]
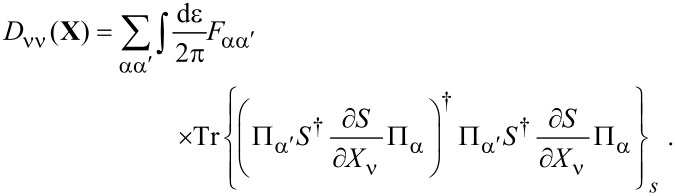


which is evidently positive.

**Damping matrix:** So far, we were able to express quantities in terms of the frozen S-matrix only. This is no longer the case for the first correction to the strictly adiabatic approximation, given by Equation 31 and Equation 32. We start here with the first of these terms, the symmetric matrix γ*^s^*, which is responsible for dissipation of the mechanical system into the electronic bath.

The manipulations to write the dissipation term as a function of S-matrix quantities are lengthy and the details are given in Supporting Information File 1, Section B. The damping matrix can be split into an “equilibrium” contribution, γ*^s,eq^*, and a purely nonequilibrium contribution γ*^s,ne^*, as γ*^s^* = γ*^s,eq^* + γ*^s,ne^*. We first treat γ*^s,eq^*. By the calculations given in Supporting Information File 1, Section B, we obtain

[46]
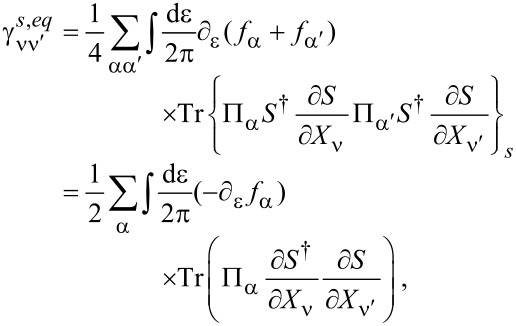


where we have used ∑_α′_Π_α′_ = 1, *S*^†^*S* = **1**, and Equation 38 in the last line. Note that in general, γ*^s,eq^* also contains nonequilibrium contributions, but gives the only contribution to the damping matrix when in equilibrium. Equation 46 is analogous to the S-matrix expression obtained for dissipation in ferromagnets in thermal equilibrium, dubbed Gilbert damping [35].

To express γ*^s,ne^* in terms of S-matrix quantities, we have to make use of the A-matrix defined in Equation 37. Again the details are given in Supporting Information File 1, Section B, where we find, after lengthy manipulations, that

[47]
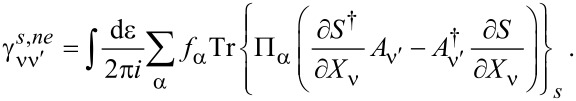


This quantity vanishes in equilibrium, as can be shown by using the properties of the *S* and *A* matrices. Since the sum over the leads can be directly performed in equilibrium, Expression 45 involves

[48]
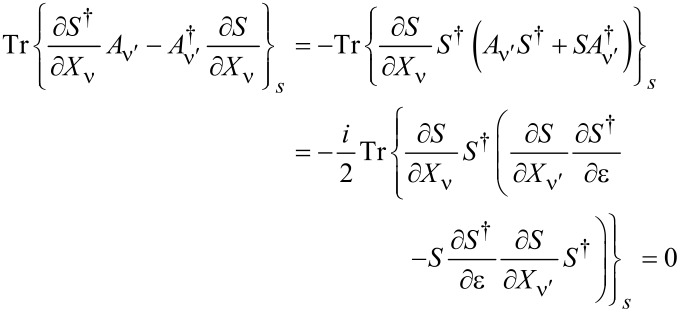


in which we have used the unitarity of 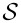 and the cyclic invariance of the trace multiple times. In the first equality, we inserted *S*^†^*S* = 1 and used Equation 38; the second equality follows by inserting the identity (Equation 40) and using again Equation 38.

Finally, combining all terms, we obtain an S-matrix expression for the full damping matrix γ*^s^*,

[4]
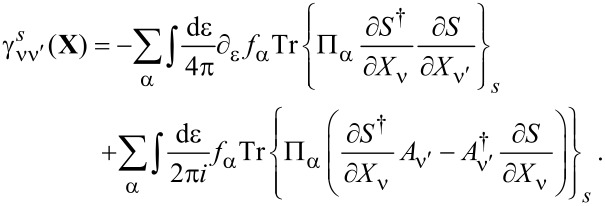


Note that in equilibrium, by the relation −*∂*_ε_* f* = *f*(1 − *f*)/*T* and using Equation 38, the fluctuating force *D* and damping γ*^s^* are related via

[49]
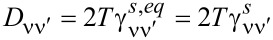


as required by the fluctuation–dissipation theorem.

Following a similar set of steps as shown above for the variance *D*_νν′_ in Equation 45, 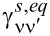 has positive eigenvalues. On the other hand, the sign of 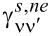 is not fixed, allowing the possibility of negative eigenvalues of γ*^s^*. The possibility of negative damping is, therefore, a pure nonequilibrium effect. Several recent papers have demonstrated negative damping in specific out-of-equilibrium models [22,40,50–51].

**Lorentz force:** We turn now to the remaining term, the antisymmetric contribution γ*^a^* given in Equation 32, which acts as an effective magnetic field. Using Equation 88 in Supporting Information File 1, Section A, it can be written as

[50]
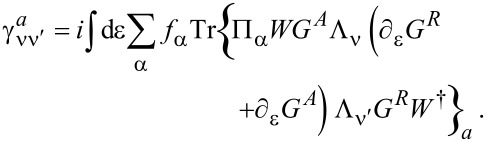


In order to relate this to the scattering matrix, we use Equation 96 (Supporting Information File 1, Section A), which allows us to write γ*^a^* in terms of the S-matrix as

[5]
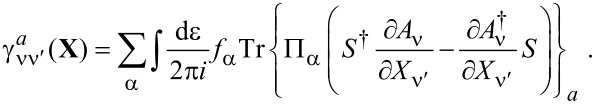


If the system is time-reversal invariant, γ*^a^* vanishes in thermal equilibrium. This implies ∑_α_Π_α_*f*_α_ = *f*, such that Equation 5 involves only


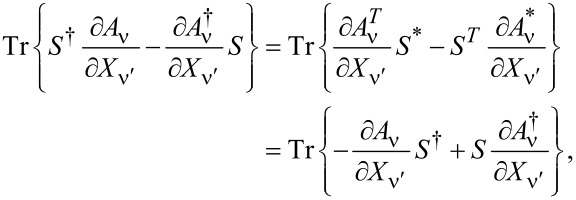


yielding γ*^a^* = 0 due to the cyclic invariance of the trace. In the last equality, we have used *S* = *S^T^* and *A* = −*A^T^* as implied by time-reversal invariance.

Out of equilibrium, γ*^a^* generally does not vanish even for time-reversal-symmetric conductors, since the current effectively breaks time-reversal symmetry.

### Current

So far we have focused on the effect of the electrons on the mechanical degrees of freedom. For a complete picture, we also need to consider the reverse effect of the mechanical vibrations on the electronic current. In the strictly adiabatic limit, this obviously has to reduce to the Landauer–Büttiker formula for the transport current. The leading adiabatic correction to the current in equilibrium is closely related to the phenomenon of quantum pumping, and we will see that our results in this limit essentially reduce to Brouwer’s S-matrix formula for the pumping current [5]. Our full result is, however, more general since it gives the leading adiabatic correction to the current in arbitrary *nonequilibrium* situations [8].

The current through lead α is given by [43]:

[51]
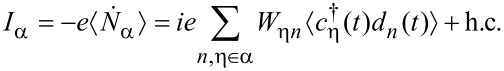


with 
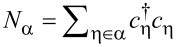
. Using the expressions for the self-energies this can be expressed in terms of the dot’s Green’s functions and self-energies,

[52]
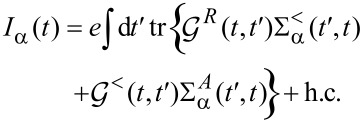


Again we use the separation of time scales and go to the Wigner representation, yielding

[53]
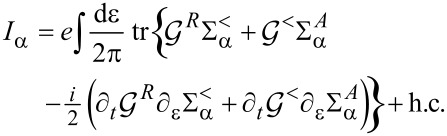


We split the current into an adiabatic contribution 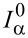 and a term proportional to the velocity 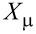:

[54]
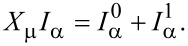


We will express these quantities in terms of the scattering matrix.

#### Landauer–Büttiker current

The strictly adiabatic contribution to the current is given by

[55]
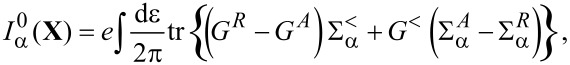


in which we have collected the purely adiabatic terms from Equation 25 and Equation 29. Inserting the expressions for the self-energies, Equation 19 and Equation 28, we can express this as

[56]
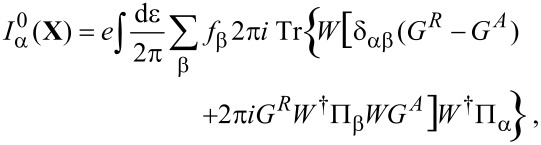


in which we have used Equation 88 (Supporting Information File, Section A). Inserting the adiabatic S-matrix, Equation 34 yields

[57]
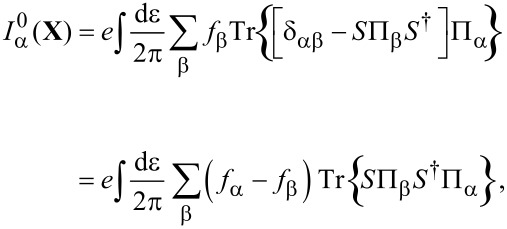


where we used ∑_β_*S*Π_β_*S*^†^ = 1 in the last line. We hence recover the usual expression for the Landauer–Büttiker current [4]. Note that the total adiabatic current depends implicitly on time through **X**(*t*), and is conserved at every instant of time, 
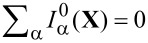
. To obtain the *direct* current, we need to average this expression over the Langevin dynamics of the mechanical degrees of freedom. Alternatively, we can average the current expression with the probability distribution of **X**, which can be obtained from the corresponding Fokker–Planck equation. Similar considerations would apply to calculations of the current noise.

#### First-order correction

We now turn to the first-order correction to the adiabatic approximation [8], restricting our considerations to the wide-band limit. The contribution to the current (Equation 53), which is linear in the velocity, reads

[58]
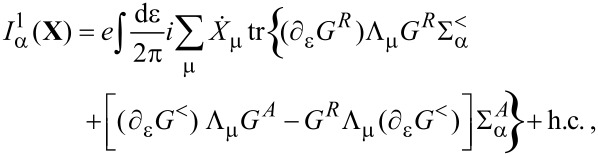


after integration by parts. Again, we insert Equation 88 from Supporting Information File 1, Section A, for the lesser Green’s function, and Equation 19, and Equation 28 for the self-energies. In the wide-band limit, the identity (*i*/2)*∂*_ε_*∂_X_*_ν_*S* + *A*_ν_ = *W*(*∂*_ε_*G^R^*)Λ_ν_*G^R^W*^†^ holds, such that we can write

[59]
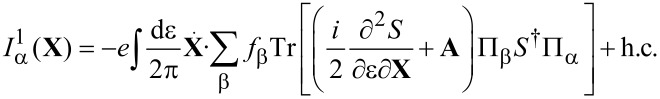


after straightforward calculation. After integration by parts, we can split this expression as

[60]
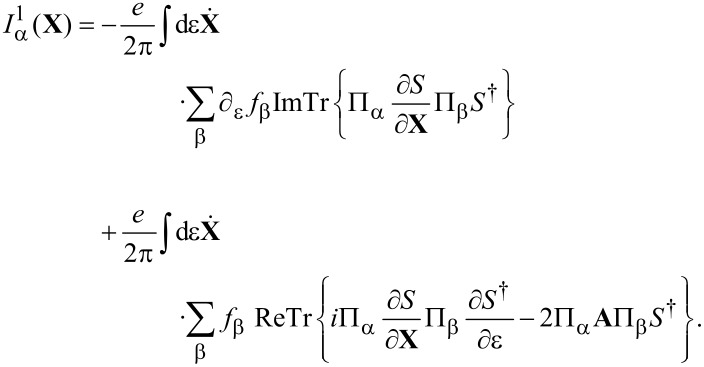


In equilibrium, the second term vanishes due to the identity in Equation 40, and the first term agrees with Brouwer’s formula for the pumping current [5]. As for the strictly adiabatic contribution, the *direct* current is obtained by averaging over the probability distribution of **X**.

### Applications

#### Resonant Level

To connect with the existing literature, as a first example we treat the simplest case within our formalism: A resonant level coupled to a single vibrational mode and attached to two leads on the left (*L*) and right (*R*). This model has been discussed in detail for zero temperature in [28–29], and it provides a simple description on how current-induced forces can be used to manipulate a molecular switch. Here we derive finite-temperature expressions for the current-induced forces for a generic coupling between electronic and mechanical degrees of freedom, starting from the scattering matrix of the system, and show how they reduce to the known results for zero temperature and linear coupling.

We consider *N* = *M* = 1, denoting the mode coordinate by *X*, the energy of the dot level by 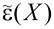, and the number of channels in the left and right leads by *N_L_* and *N_R_*, respectively. The Hamiltonian of the dot can then be written as

[61]
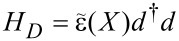


and the hybridization matrix as *W*^†^ = (**w***^L^*,**w***^R^*)^†^, with 
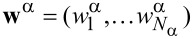
 and α = *L*,*R*. Hence the frozen S-matrix, Equation 34, is given by

[62]
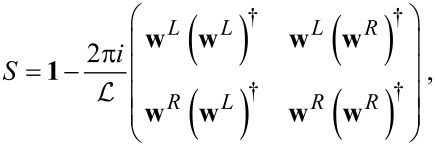


where 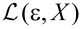 = 
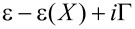
, Γ = Γ*_L_* + Γ*_R_*, and Γ_α_ = π(**w**^α^)^†^·**w**^α^. Rotating to an eigenbasis of the lead channels, this S-matrix does not mix channels within the same lead, and hence we can project the S-matrix into a single nontrivial channel in each lead, to obtain

[63]
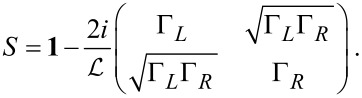


To calculate the mean force from Equation 2, we need an explicit expression for Equation 93 in Supporting Information File 1, Section A. This can be easily calculated to be

[64]
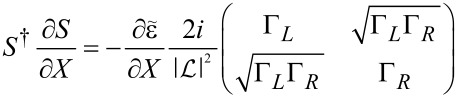


and hence

[65]
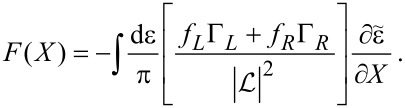


Analogously, the variance of the stochastic force, Equation 3, becomes

[66]
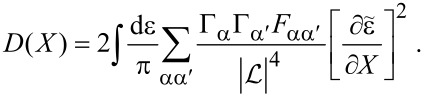


All that remains is to calculate the dissipation coefficient γ. Since there is only one collective mode, ν = 1, γ is a scalar and hence γ*^a^* = 0. Moreover, for energy-independent hybridization we have 
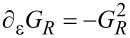
, and the A-matrix (Equation 37) can be written as [22]

[67]
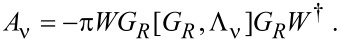


Being the commutator of scalars, in this case *A*_1_ = 0 and from Equation 4, γ*^s^* must be positive and is given by Equation 46. (For an alternative derivation confirming the positive sign of the friction coefficient in a resonant-level system, see [52]). After some manipulation, we obtain

[68]
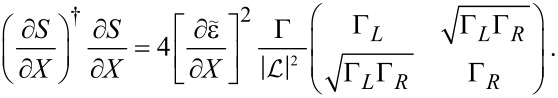


and hence the damping coefficient becomes

[69]
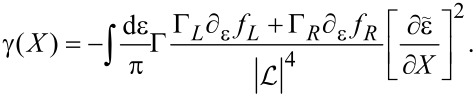


We can evaluate the remaining integrals analytically in the zero-temperature limit [28–29]. In the following we assume that μ*_L_* ≥ μ*_R_*. The average force is given by

[70]
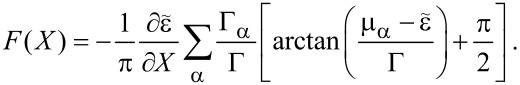


Similarly we obtain the dissipation coefficient

[71]
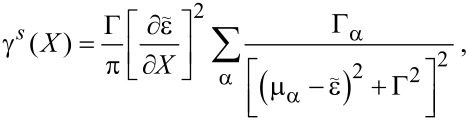


together with the fluctuation kernel

[72]
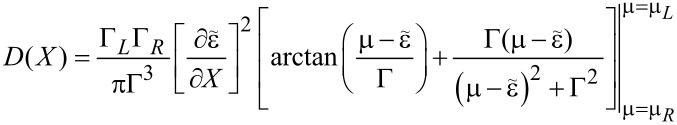


The position of the dot electronic level can be adjusted by an external gate voltage

[73]
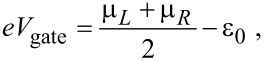


where the factor (μ*_L_* + μ*_R_*)/2 is included for convenience, in order to measure energies from the center of the conduction window. The difference in chemical potential between the leads is adjusted by a bias voltage

[74]
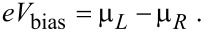


**Figure 1:**
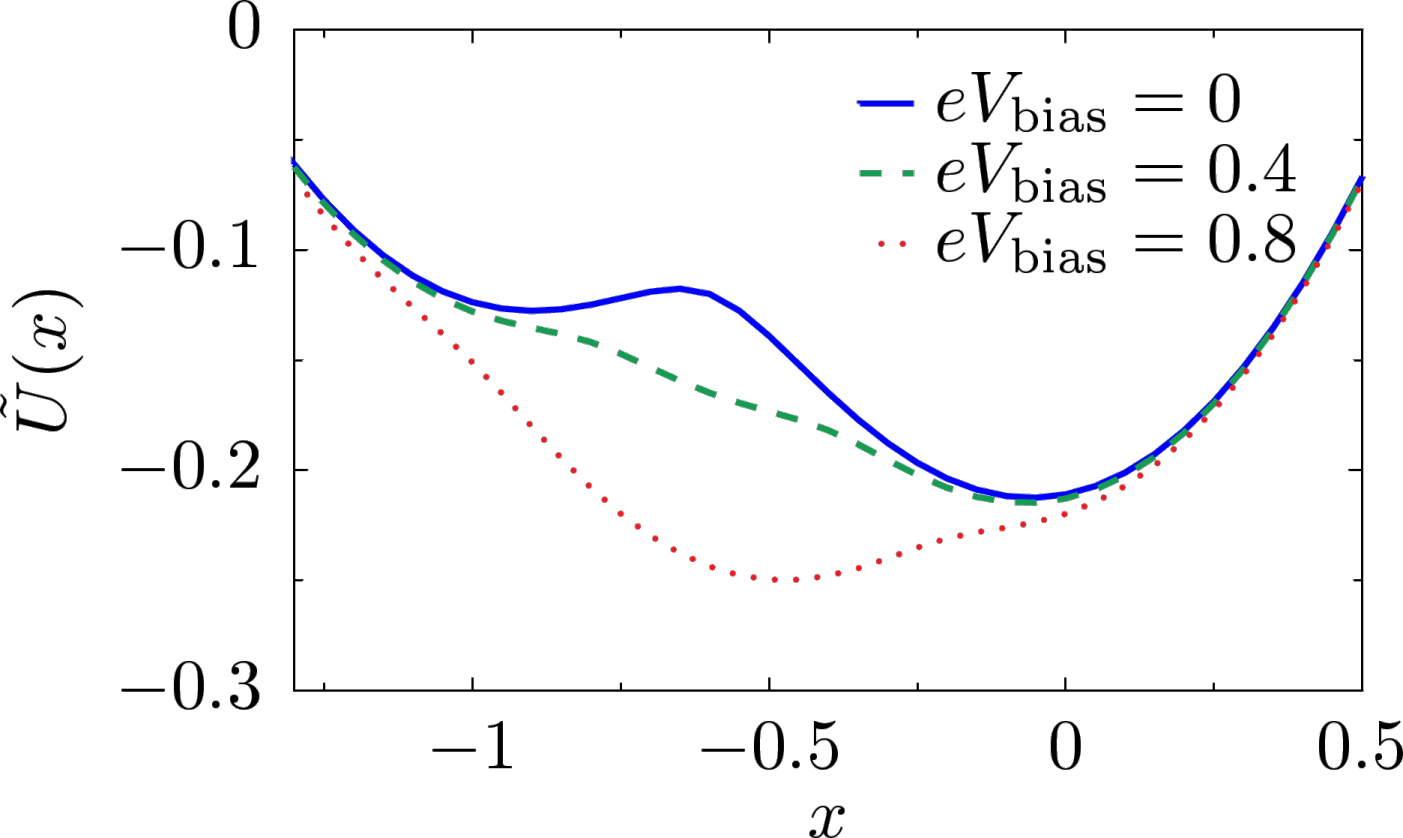
Resonant level. The shape of the effective potential 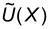 can be tuned by the bias voltage. We consider the parameters *eV*_gate_ = 0, 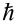
ω_0_ = 0.01 and Γ = 0.1. The dimensionless coordinate is *x* = (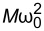/*λ*)*X* and energies are measured in units of λ^2^/(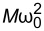).

For a single vibrational mode, the average current-induced force is necessarily conservative and we can define a corresponding potential. Restricting our results now to linear coupling, we write the local level as 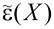 = ε_0_ + λ*X*. In Figure 1, we show the effective potential 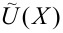 = 
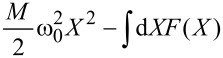
, which describes both the elastic and the current-induced forces at zero temperature and various bias voltages. Already this simple example shows that the current-induced forces can affect the mechanical motion qualitatively [29]. Indeed, the effective potential 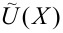 can become multistable even for a purely harmonic elastic force, and depends sensitively on the applied bias voltage.

Alternative expressions for the current-induced forces for the resonant-level model, in terms of phase shifts and transmission coefficients, are given in Supporting Information File 1, Section C.

#### Two-level model

**Figure 2:**
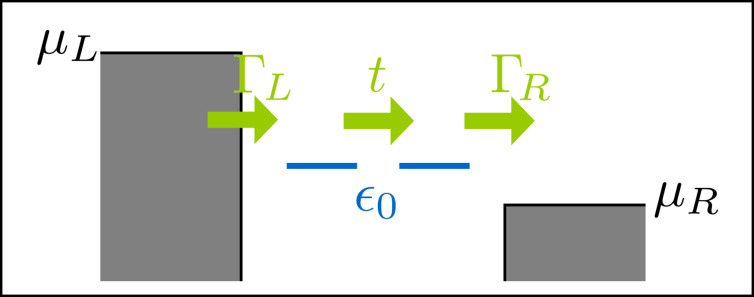
Sketch of the two-level model. Electrons tunnel through two degenerate energy levels between the left and right leads. The system is modulated by the coupling to the vibrational modes.

For the resonant-level model discussed so far, the A-matrix vanishes and the damping is necessarily positive. We now consider a model that allows for negative damping [53]. Our toy model can be seen to be inspired by a double dot on a suspended carbon nanotube, or an H_2_ molecule in a break junction. The model is depicted schematically in Figure 2. The bare dot Hamiltonian corresponds to degenerate electronic states ε_0_, localized on the left and right atoms or quantum dots, with tunnel coupling *t* in between,

[75]
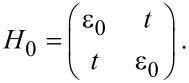


We consider a single oscillator mode, with coordinate *X*, that couples linearly to the difference in the occupation of the levels. In our previous notation, this means that Λ_1_ = λ_1_σ_3_, where σ_μ_, with μ = 0,…, 3, denotes the Pauli matrices acting in the two-site basis. The shift of the electronic levels is given by 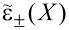 = ε_0_ ± λ_1_*X*.

The hybridization matrices are given by Γ^α^ = (1/2)Γ_α_(σ^0^ ± σ^3^), where the +(−) refers to α = *L*(*R*). We can deduce the tunneling matrix *W* in terms of the hybridization matrices,

[76]
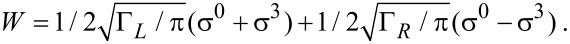


In the wide-band limit, we approximate *W* and Γ_α_ to be independent of energy. The retarded adiabatic GF takes the form

[77]
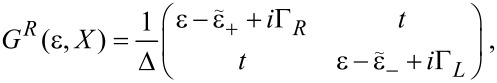


with 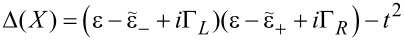.

For simplicity, we restrict our attention to symmetric couplings to the leads, Γ*_L_* = Γ*_R_* = Γ/2. Hence the frozen S-matrix *S*(ε*,X*) becomes

[78]
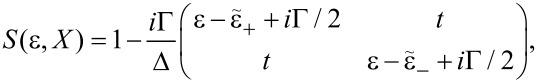


while the A-matrix takes the form

[79]
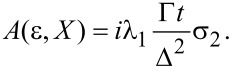


We can now give explicit expressions for the current-induced forces. The explicit expressions are lengthy and are given in Equation 115 and Equation 116 (Supporting Information File 1, Section D) for the mean force and damping matrix, respectively. The variance of the fluctuating force can be calculated accordingly.

**Figure 3:**
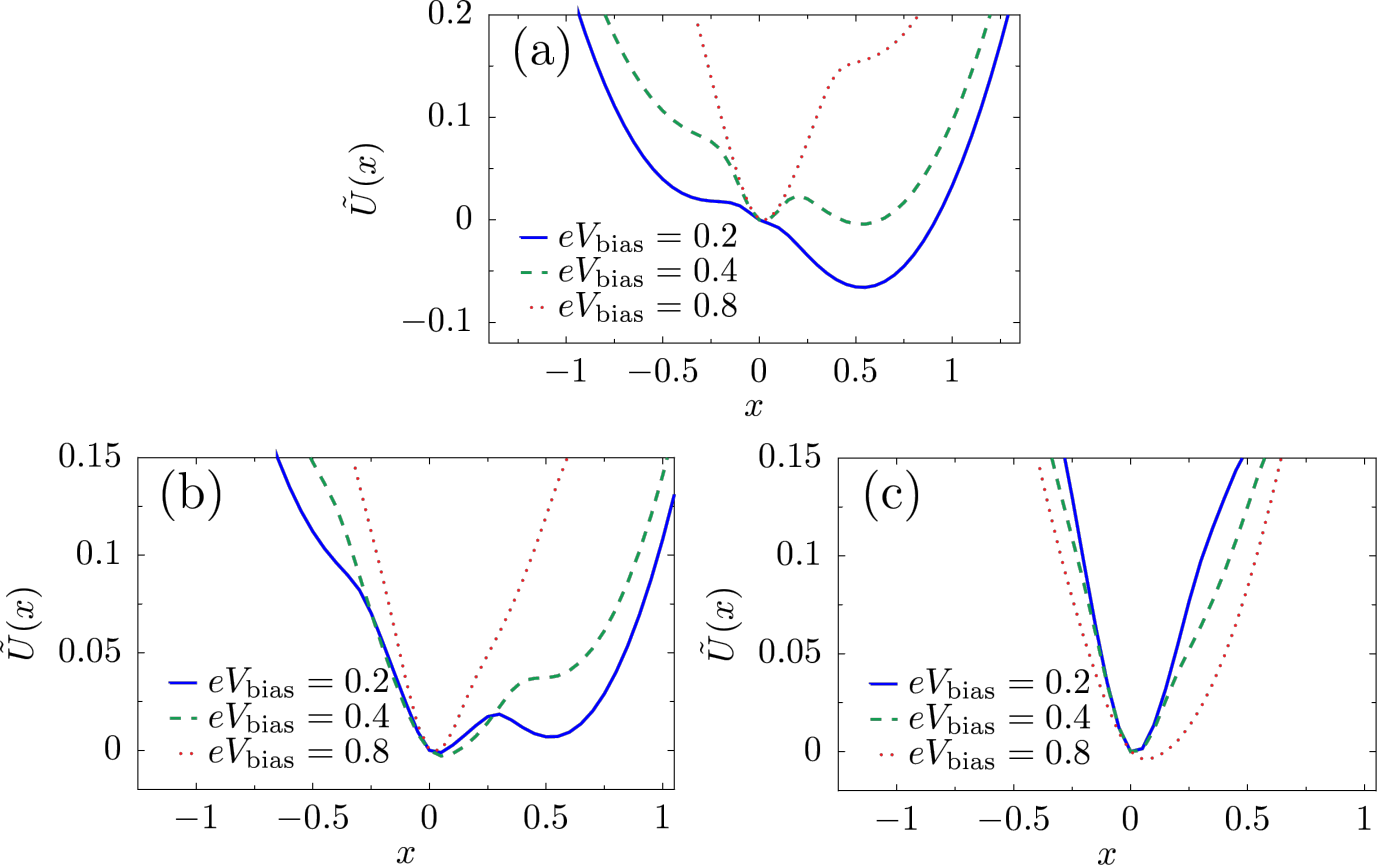
Effective potential for the mechanical motion in the two-level model. The shape of the potential can be tuned by changing the bias and gate voltages: (a) *eV*_gate_ = 0, (b) *eV*_gate_ = 0.2 and (c) *eV*_gate_ = 0.4. We consider the parameters 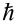
ω_0_ = 0.01, *t* = 0.1 and Γ = 0.1. The dimensionless coordinate is *x* = (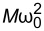/λ_1_)*X* and energies are measured in units of 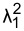/(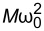).

The average force given in Equation 115 (Supporting Information File 1, Section D) combines with the elastic force to give rise to the effective potential 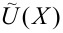 depicted, for zero temperature, in Figure 3. As in the case studied in the previous section, the system can exhibit various levels of multistability with changes in the bias.

**Figure 4:**
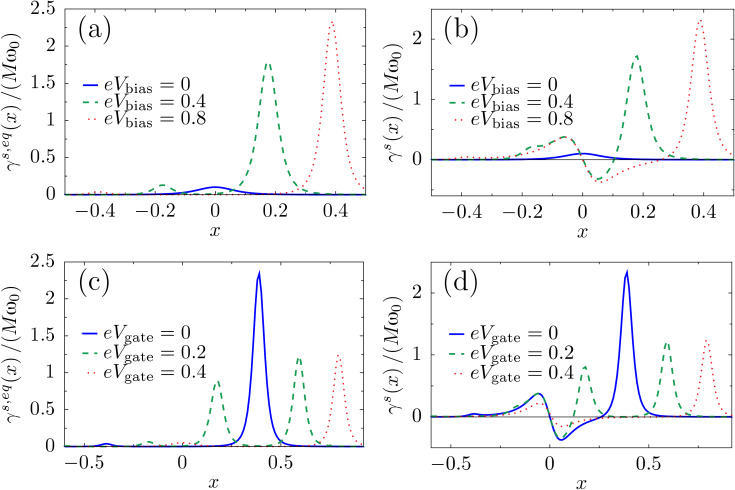
Damping versus mechanical displacement in the two-level model. (a) Contribution γ*^s,eq^* to the friction coefficient for various bias voltages at fixed gate voltage *eV*_gate_ = 0. (b) At the same gate voltage, the total damping exhibits a region of negative damping due to the contribution of γ*^s,ne^*. (c) γ*^s,eq^* for various gate voltages with the bias voltage *eV*_bias_ = 0.8. Note that for both *eV*_gate_ = 0.2 and *eV*_gate_ = 0.4, one small peak for negative *x* falls outside of the range of *x* shown. (d) Again, the full damping γ*^s^* exhibits regions of negative damping. We choose 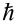
ω_0_ = 0.01, Γ = 0.1 and *t* = 0.1. The dimensionless coordinate is *x* = (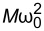/λ_1_)*X* and energies are measured in units of 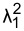/(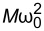).

**Figure 5:**
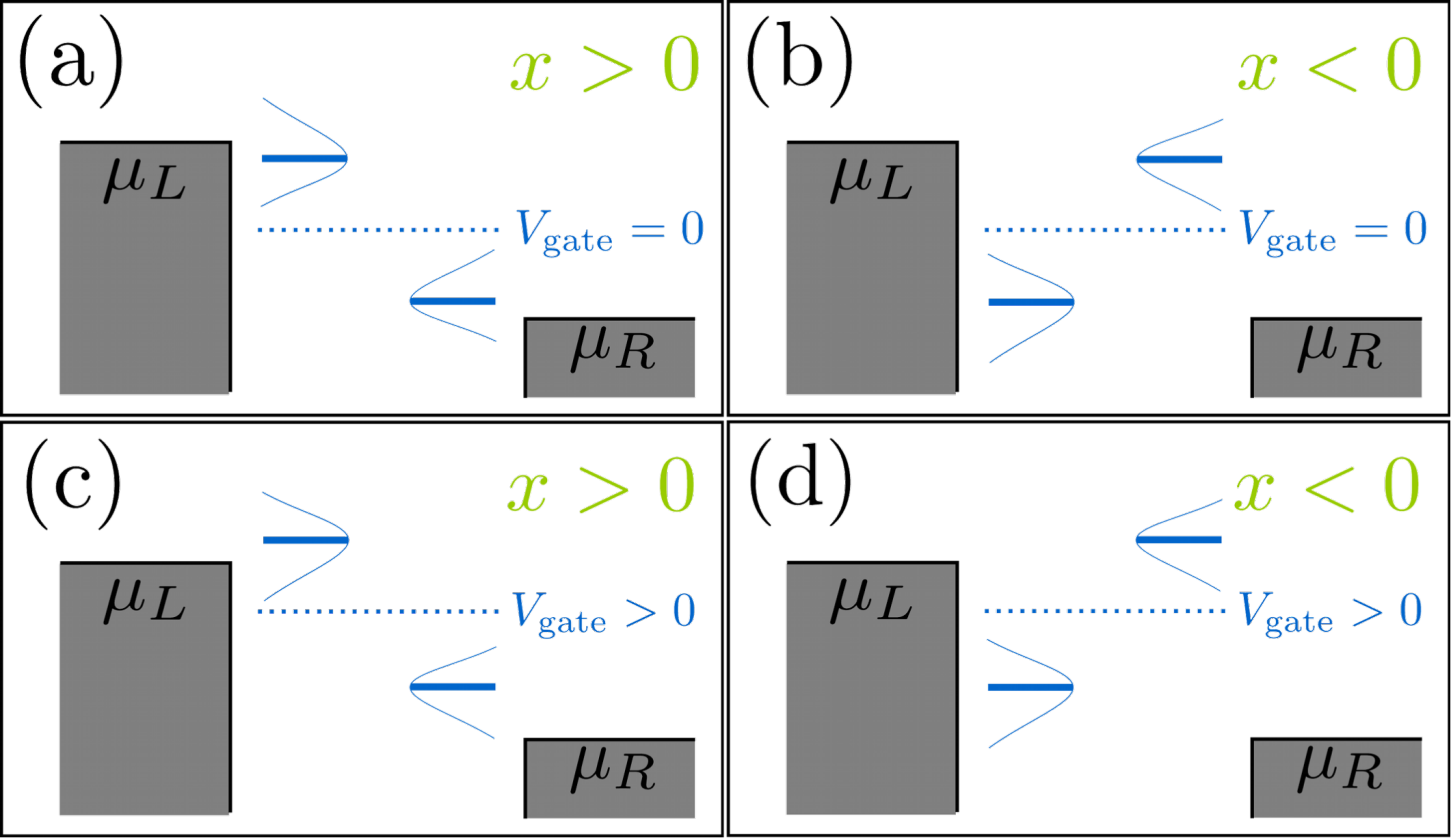
Cartoon of the positions of the electronic levels in the dot with respect to the Fermi levels of the leads, depending on the sign of *x* and the existence of a gate voltage. The levels are broadened due to the hybridization with the leads, Γ. When *x* > 0, “left” and “right” levels approach the Fermi levels of the left and right leads respectively; (a) for *eV*_gate_ = 0 the levels align simultaneously for left and right; (c) a finite *eV*_gate_ produces an asymmetry between left and right. For *x* < 0 the alignment of the levels is inverted; (b) *eV*_gate_ = 0; (d) finite *eV*_gate_.

The results for the friction coefficient, given in Equation 116 (Supporting Information File 1, Section D), are shown in Figure 4 as a function of the dimensionless oscillator coordinate *x*, for zero temperature. The contribution γ*^s,eq^* to the friction coefficient is peaked at *eV*_gate_ ± *eV*_bias_/2 = 
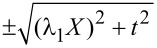
, as depicted in Figure 4a and Figure 4c. Neglecting the coupling to the leads, our toy model can be considered as a two-level system with level-spacing 
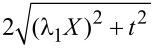
. Thus, the peaks occur when one of the electronic levels of the dot enters the conduction window. When this happens, small changes in the oscillator coordinate *X* can have a large impact on the occupation of the levels. This effect is more pronounced when the levels of the dots pass the Fermi levels that they are directly attached to [corresponding to *X* > 0 for current flowing from left to right, see Figure 4a, Figure 5a, and Figure 5b]. The broadening of the peaks is due to the hybridization with the leads, Γ/2. When *eV*_gate_ = 0, two peaks are expected symmetrically about *X* = 0, as shown in Figure 4a [see also Figure 5a and Figure 5b]. The effect of a finite gate voltage *eV*_gate_ is two-fold: It shifts the noninteracting electronic levels of the dot away from the middle of the conduction window, and hence the shifted levels 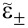 pass the Fermi levels of the right and left leads at different values of *X*, Figure 5c and Figure 5d. Therefore in this case four peaks are expected, with two larger peaks located at *X* > 0, and two smaller peaks located at *X* < 0. This is shown in Figure 4c. The height of the peaks in this case is reduced with respect to the case *eV*_gate_ = 0, since for a given peak, only one of the levels of the dot is in resonance with one of the leads. Note that four real values of *X* can be obtained only if (*eV*_gate_ ± *eV*_bias_/2)^2^ > *t*^2^. A situation with (*eV*_gate_ − *eV*_bias_/2)^2^ < *t*^2^ while (*eV*_gate_ + *eV*_bias_/2)^2^ > *t*^2^ is shown in Figure 4c (red-dotted line), in which a large peak is observed for *X* = 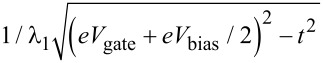, as well as a corresponding small peak for *X* = 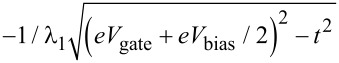 [not displayed in Figure 4c], and a peak at *X* = 0.

**Figure 6:**
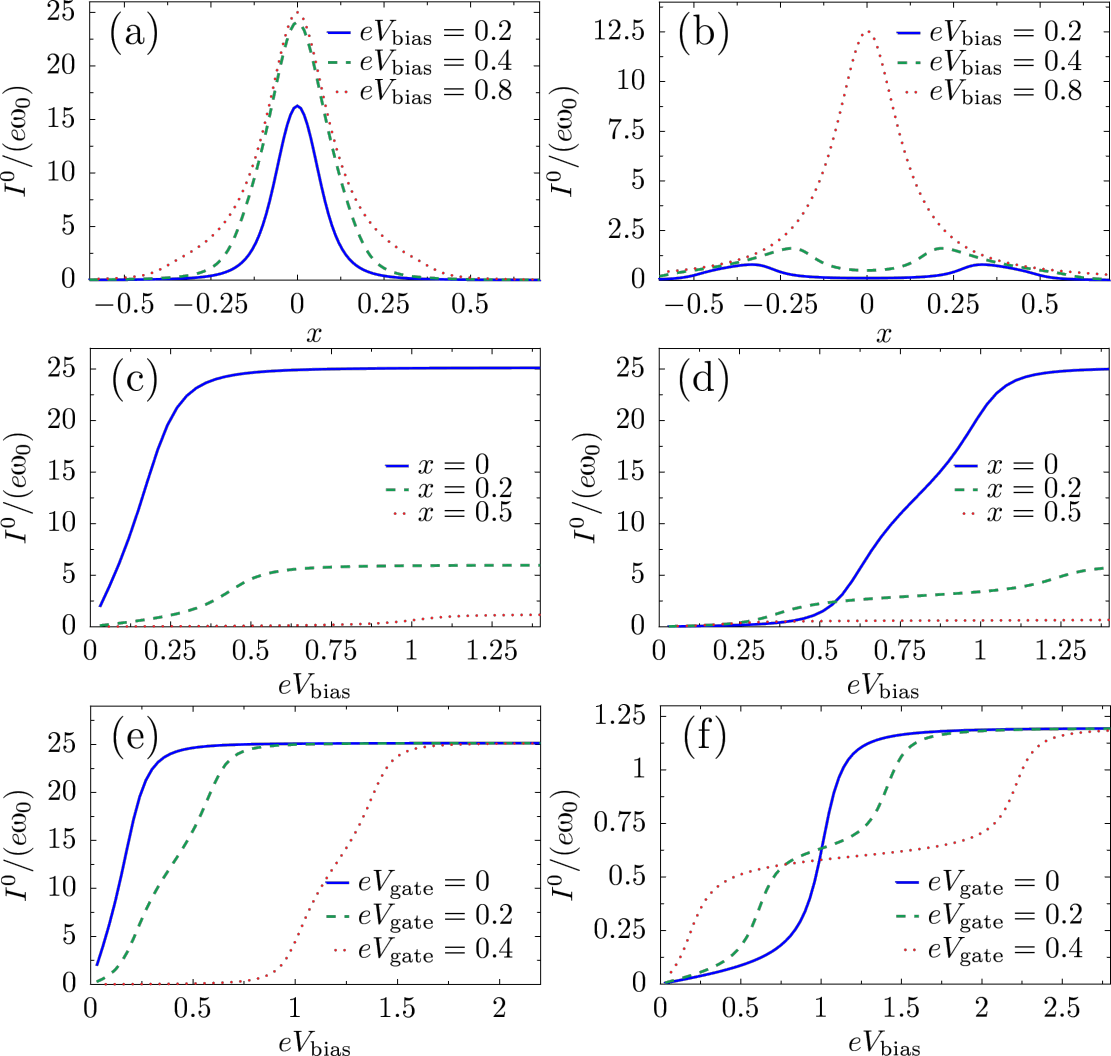
Dependence of the current in the two-level model on various parameters. Current as a function of mechanical displacement for (a) *V*_gate_ = 0 and (b) *V*_gate_ = 0.4; as function of bias for (c) *V*_gate_ = 0, (d) *V*_gate_ = 0.4, (e) *x* = 0 and (f) *x* = 0.5. We choose 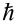
ω_0_ = 0.01, Γ = 0.1 and *t* = 0.1. The dimensionless coordinate is *x* = (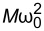/*λ*_1_)*X* and energies are measured in units of 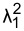/(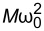).

For this model, the A-matrix is generally nonvanishing, which can result in negative damping for out-of-equilibrium situations. This is due to a negative contribution of γ*^s,ne^* to the total damping. This is visualized in Figure 4b and Figure 4d. Negative damping is possible when both dot levels are inside the conduction window, restricting the region in *X* over which negative damping can occur. Indeed, when only one level is within the conduction window, the system effectively reduces to the resonant level model for which, as we showed in the previous subsection, the friction coefficient γ*^s^* is always positive. When current flows from left to right, negative damping occurs only for positive values of the oscillator coordinate *X*, as shown in Figure 4b and Figure 4d. This is consistent with a level-inversion picture, as discussed recently in [51]. Pictorially, the electron–vibron coupling causes a splitting in energy of the left and right levels. When *X* > 0, electrons can go “down the ladder” formed by the energy levels by passing energy to the oscillator and hence amplifying the vibrations. For *X* < 0, electrons can pass between the two dots only by absorbing energy from the vibrations, causing additional nonequilibrium damping. For small broadening of the dot levels due to the coupling to the leads, this effect is expected to be strongest when the vibration-induced splitting λ_1_*X* becomes of the same order as the strength of the hopping *t*. When *X* grows further, the increasing detuning of the dot levels reduces the current and hence the nonequilibrium damping [Figure 4b and Figure 4, and below in Figure 6]. The coexistence of a multistable potential together with regions of negative damping can lead to interesting nonlinear behavior for the dynamics of the oscillator. In particular, and as we show in the next example, limit-cycle solutions are possible, in the spirit of a Van der Pol oscillator [54].

We can also calculate the current. The pumping contribution is proportional to the velocity 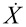 and thus small. Therefore we show here results only for the dominant adiabatic part of the current. This is given by

[80]
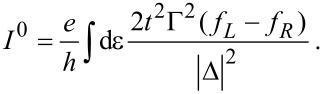


For zero temperature, the behavior of the current is shown in Figure 6 as a function of various parameters. Figure 6a and Figure 6b show the current as a function of the (dimensionless) oscillator coordinate *x* for two different values of gate potential for which the system exhibits multistability by developing several metastable equilibrium positions. For *V*_gate_ = 0, and independently of bias, the current shows a maximum at the local minimum of the effective potential *x* = 0, while *I*^0^ ≈ 0 for another possible local minimum, *x* ≈ 0.5 (compare with Figure 3a). The true equilibrium value of *x* can be tuned through the bias potential, offering the possibility of perfect switching. For finite gate potential, however, the current is depleted from *x* = 0 with diminishing bias. Figure 6c and Figure 6d show the current as a function of gate or bias voltage for fixed representative values of the oscillator coordinate *x*. The current changes stepwise as the number of levels inside the conduction window changes, coinciding with the peaks in the friction coefficient illustrated in Figure 4. In an experimental setting, the measured *direct* current would involve an average over the probability distribution of the coordinate *x*, given by the solution of the Fokker–Planck equation associated with the Langevin Equation 1.

#### Two vibrational modes

As a final example, we present a simple model that allows for both a nonconservative force and an effective “Lorentz” force, in addition to negative damping. For this it is necessary to couple the two electronic orbitals of the previous example, see Equation 75, to at least two oscillatory modes that we assume to be degenerate. The relevant vibrations in this case can be thought of as a center-of-mass vibration *X*_1_ between the leads, and a stretching mode *X*_2_. (It should be noted that this is for visualization purposes only. In reality, for an H_2_ molecule, the stretching mode is a high energy mode when compared to a transverse and a rotational mode [55]. Nevertheless, the H_2_ molecule does indeed have two near-degenerate low-energy vibrational modes, corresponding to rigid vibrations between the leads and a rigid rotation relative to the axis defined by the two leads.) The stretch mode modulates the hopping parameter,

[81]
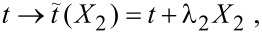


while the center of mass mode *X*_1_ is modeled as being coupled linearly to the density,

[82]
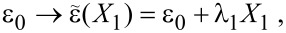


hence Λ_1_ = λ_1_σ_0_ and Λ_2_ = λ_2_σ_1_. We work in the wide-band limit, but allow for asymmetric coupling to the leads. The retarded Green’s function becomes

[83]
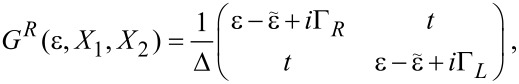


where now 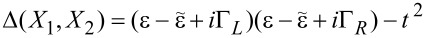. The frozen S-matrix can be easily calculated to be

[84]
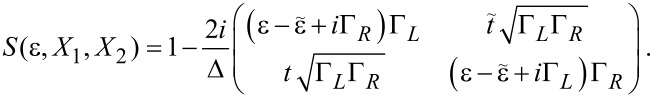


The A-matrices also take a simple form for this model. Since Λ_1_ is proportional to the identity operator,

[85]
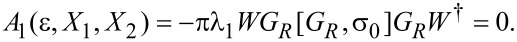


On the other hand, the A-matrix associated with *X*_2_ is nonzero and given by

[86]
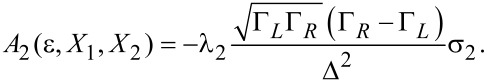


From this we can compute the average force, damping, pseudo-Lorentz force, and noise terms. These are listed in Supporting Information File 1, Section E. At zero temperature, it is possible to obtain analytical expressions for these current-induced forces. Studying the dynamics of the modes *X*_1,2_(*t*) implies solving the two coupled Langevin equations given by Equation 1, after inserting the expressions for the forces given in Supporting Information File 1, Section E. Within our formalism we are able to study the full nonlinear dynamics of the problem, which brings out a plethora of new qualitative behavior. In particular, analyses that linearize the current-induced force about a static-equilibrium point would predict run-away modes due to negative damping and nonconservative forces [30]. Taking into account nonlinearities allows one to find the new stable attractor of the motion. Indeed, we find that these linear instabilities typically result in dynamic equilibrium, namely limit-cycle dynamics [22]. We note in passing that limit-cycle dynamics in a nanoelectromechanical system was also discussed recently in [53].

**Figure 7:**
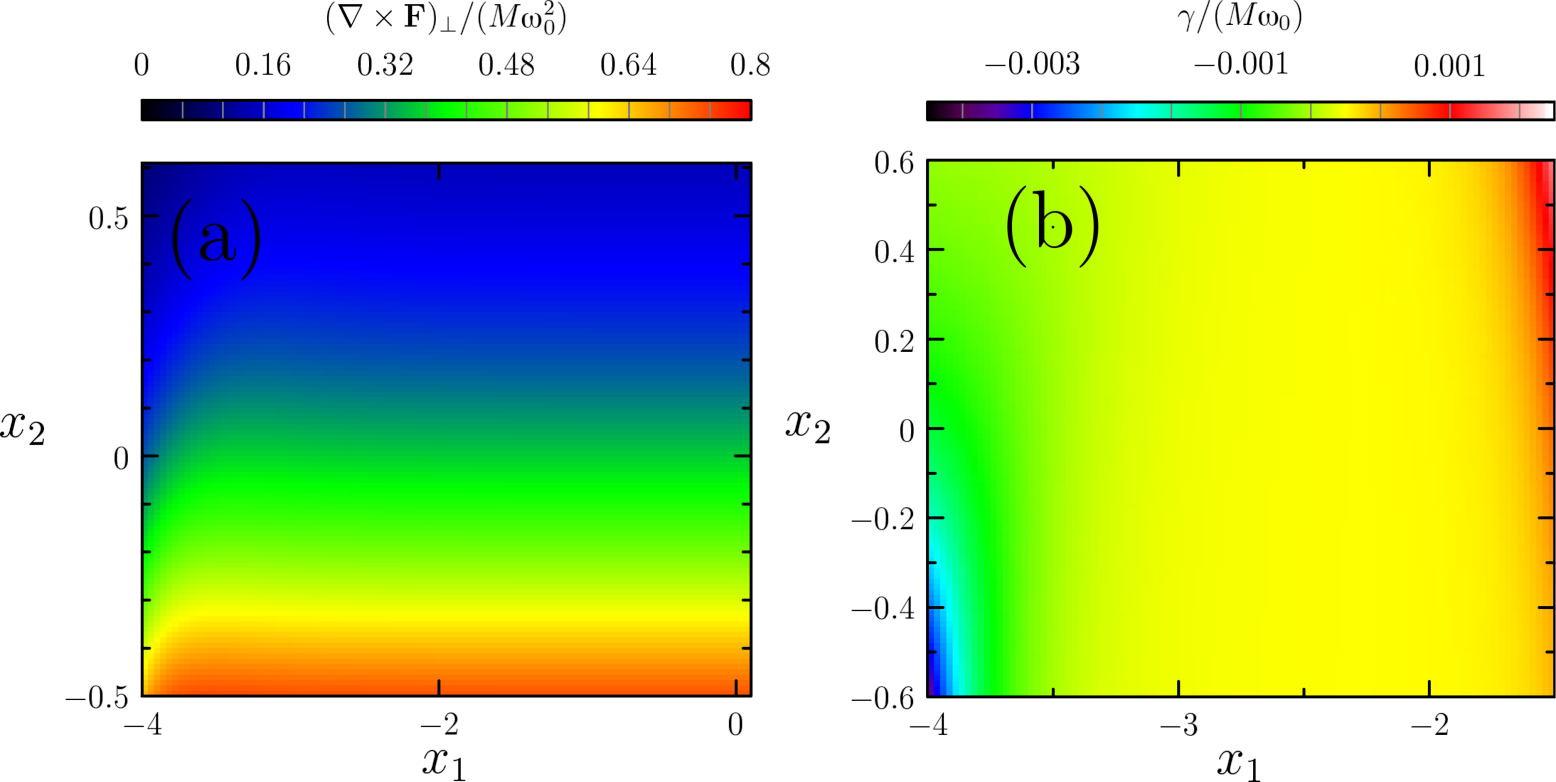
Curl of the average force and damping coefficient for the model with two vibrational modes: (a) The curl of the current-induced mean force **F** is, in a nonequilibrium situation, generally nonzero, indicating that the force is nonconservative. (b) One of the two eigenvalues of γ*^s^*. Remarkably, it undergoes changes of sign. A dissipation matrix γ*^s^* that is non-positive-definite implies destabilization of the static-equilibrium solution found at lower bias potentials, in this case driving the system into a limit cycle, see main text and Figure 8. The parameters used are such that λ_1_/λ_2_ = 3/2. The elastic modes are degenerate with 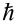
ω_0_ = 0.014, Γ*_L,R_* = [(1 ± 0.8)/2](σ_0_ ± σ_3_), and the hopping between the orbitals is *t* = 0.9. The dimensionless coordinates are *x_i_* = (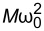/λ)*X_i_* and energies are in units of λ^2^/(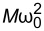), where λ = (λ_1_ + λ_2_)/2.

**Figure 8:**
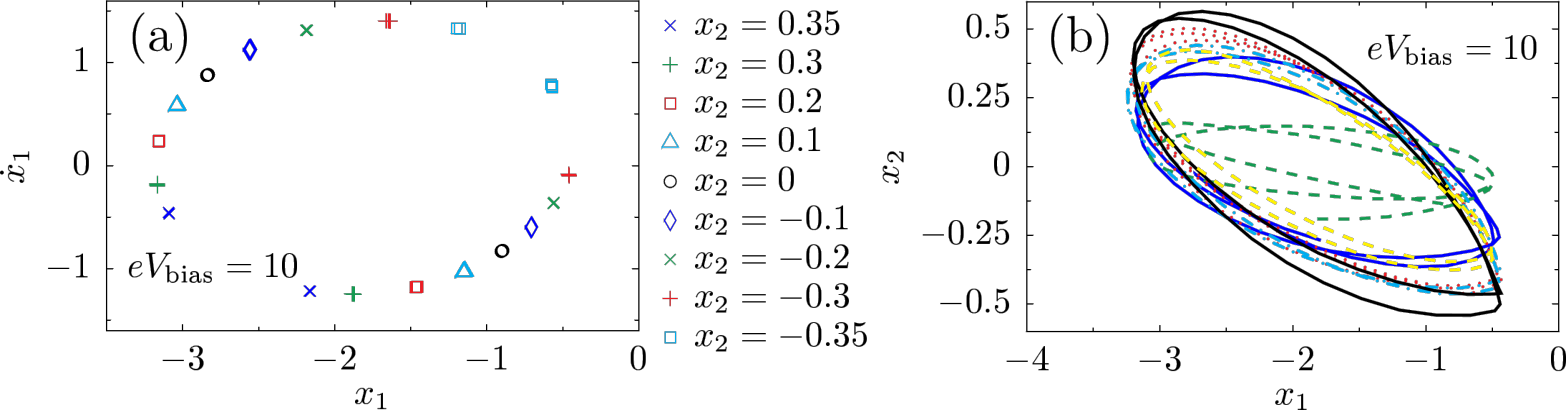
Limit-cycle dynamics for the model with two vibrational modes. (a) At large bias voltages, Poincaré sections of the four-dimensional phase space show the presence of a limit cycle in the Langevin dynamics without a fluctuating force. (b) Several periods of typical trajectories (for different initial conditions after a transient) in the presence of the fluctuating forces ξ are shown. The same general parameters as in Figure 7 are used here.

We have studied the zero-temperature dynamics of our two-level, two-mode system for different ranges of parameters. In Figure 7 we map out the values of the curl of the mean force, 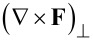, indicating that the force is nonconservative throughout parameter space. We also plot one of the two eigenvalues of the dissipation matrix γ*^s^*, showing that it can take negative values in some regions of the parameter space. We find that it is possible to drive the system into a limit cycle by varying the bias potential. The existence of this limit cycle is shown in Figure 8a, where we have plotted various Poincaré sections of the nonlinear system without fluctuations. The figure shows the trajectory in phase space of the (dimensionless) oscillator coordinate *x*_1_ after the dynamic equilibrium is reached, for several cuts of the (dimensionless) coordinate *x*_2_. Each cut shows two points in *x*_1_ phase space, indicating the entry and exit of the trajectory. Each point in the plot actually consists of several points that fall on top of each other, corresponding to every instance in which the coordinate *x*_2_ has the value indicated in the legend of Figure 8a. This shows the periodicity of the solution of the nonlinear equations of motion for *x*_1_,*x*_2_ for the particular bias chosen. Surveying the various values of *x*_2_ reveals a closed trajectory in the parametric coordinate space *x*_1_,*x*_2_. Remarkably, signatures of the limit cycle survive the inclusion of the Langevin force. Figure 8b depicts typical trajectories in the coordinate space of the oscillator, *x*_1_,*x*_2_, in the presence of the stochastic force, showing fluctuating trajectories around the stable limit cycle.

**Figure 9:**
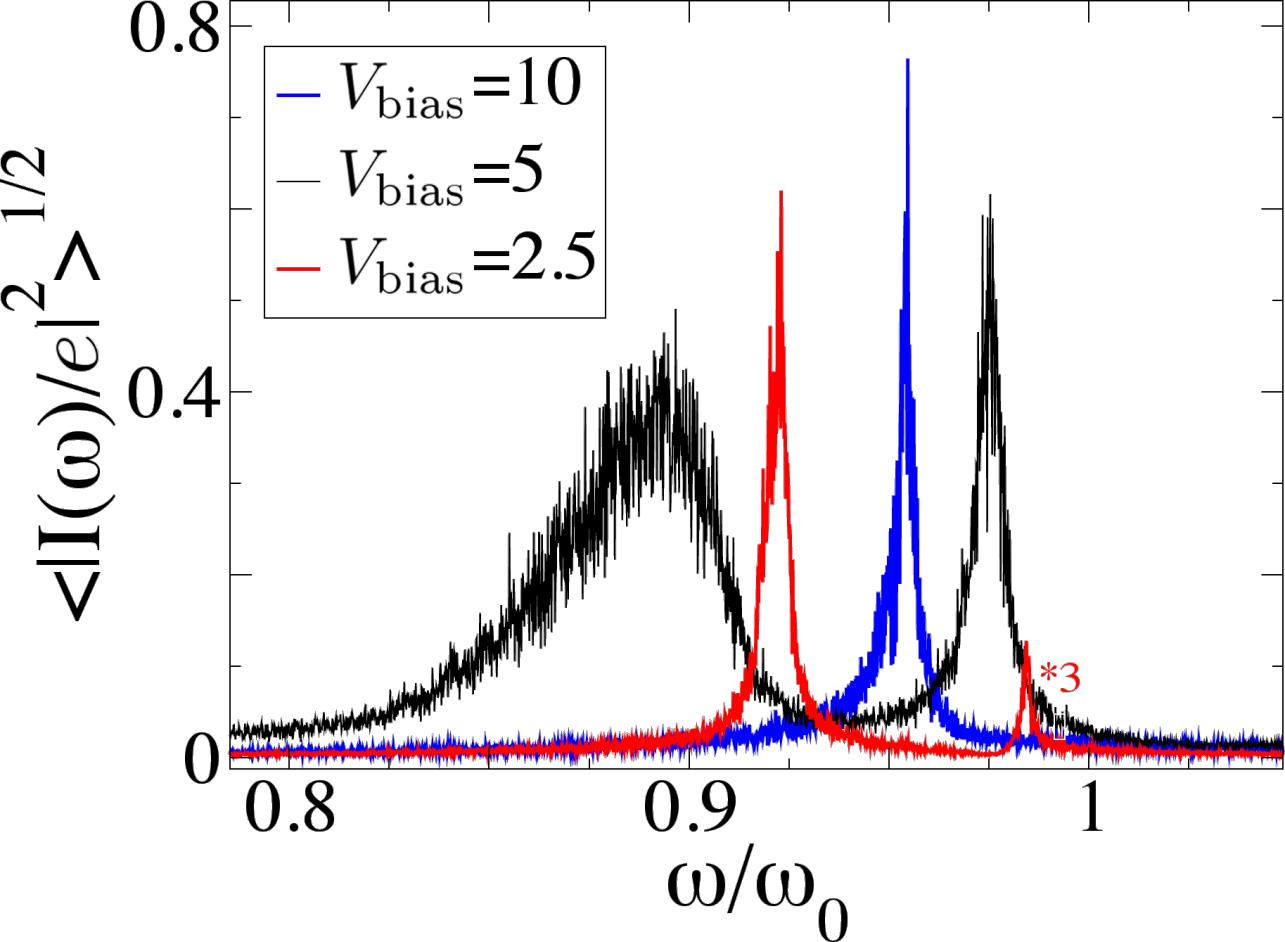
Current–current correlation function in the presence of noise for the system with two vibrational modes. The limit cycle is signaled by a single peak (*V_bias_* = 10, see Figure 8), as opposed to two peaks in the absence of a limit cycle (*V_bias_* = 2.5,5.0). Increasing the bias potential increases the noise levels, but the peaks are still easily recognizable. The results are obtained by averaging over times long enough compared with the characteristic oscillation times. The same general parameters as in Figure 7 are used here.

Experimentally, the signature of the limit cycle would be most directly reflected in the current–current correlation function, as depicted in Figure 9. We find that in the absence of a limit cycle the system is dominated by two characteristic frequencies, shown by the peaks in Figure 9. These frequencies correspond to the shift in energy of the two degenerate vibrational modes due to the average current-induced forces *F*_1_ and *F*_2_. When the bias voltage is such that the system enters a limit cycle, the current–current correlation shows instead only one peak as a function of frequency. This result, as shown in Figure 9, is fairly robust to noise, making the onset of limit-cycle dynamics observable in experiment.

## Conclusion

Within a nonequilibrium Born–Oppenheimer approximation, the dynamics of a nanoelectromechanical system can be described in terms of a Langevin equation, in which the mechanical modes of the mesoscopic device are subject to current-induced forces. These forces include a mean force, which is independent of velocity and due to the average net force that the electrons exert on the oscillator; a stochastic Langevin force, which takes into account the thermal and nonequilibrium fluctuations with respect to the mean force value; and a force linear in the velocity of the modes. This last, velocity-dependent force, consists of a dissipative term and a term that can be interpreted as an effective “Lorentz” force, due to an effective magnetic field acting in the parameter space of the modes.

In this work we have expressed these current-induced forces through the scattering matrix of the coherent mesoscopic conductor and its parametric derivatives, extending the results found previously in [22]. Our results are now valid for a generic coupling between the electrons and the vibrational degrees of freedom, given by a matrix *h*_0_(**X**), and for energy-dependent hybridization with the leads, given by the matrix *W*(ε). We have shown that expressing *all* the current-induced forces in terms of the S-matrix is only possible by going beyond the strictly adiabatic approximation, and it is necessary to include the first-order correction in the adiabatic expansion. This introduces a new fundamental quantity into the problem, the A-matrix, which needs to be calculated together with the frozen S-matrix for a given system.

There are several circumstances in which the first nonadiabatic correction, encapsulated in the A-matrix, is necessary. While the average as well as the fluctuating force can be expressed solely in terms of the adiabatic S-matrix, the A-matrix enters both the frictional and the Lorentz-like force. In equilibrium, the frictional force reduces to an expression in terms of the adiabatic S-matrix. Out of equilibrium, however, an important new contribution involving the A-matrix appears. In contrast, the A-matrix is always required in order to express the Lorentz-like force, even when the system is in thermal equilibrium.

The expressions for the current-induced forces in terms of the scattering matrix allow us to extract important properties from general symmetry arguments. Driving the nanoelectromechanical system out of equilibrium by imposing a bias results in qualitatively new features for the forces. We have shown that the mean force is nonconservative in this case, and that the dissipation coefficient acquires a nonequilibrium contribution that can be negative. We have also shown that when considering more than one mechanical degree of freedom, a pseudo-Lorentz force is present even for a time-reversal invariant system, unless one also imposes thermal equilibrium on top of the time-reversal condition.

Our model allows one to study, within a controlled approximation, the nonlinear dynamics generated by the interplay between current and vibrational degrees of freedom, opening up the path for a systematic study of these devices. By means of simple model examples, we have shown that it is possible to drive a nanoelectromechanical system into interesting dynamically stable regimes, such as a limit cycle, by varying the applied bias potential. In a limit cycle, the vibrational modes vary periodically in time, which could be the operating principle for a molecular motor. On the other hand, the possibility of nonconservative forces could also allow one to extract energy from the system, providing a controllable tool for cooling. The study of these types of phenomena in realistic systems would be an interesting application of the formalism presented in this paper.

## Supporting Information

File 1 Useful mathematical relations and detailed calculations
