# Supplementary material for: Current-induced forces in mesoscopic systems: A scattering-matrix approach
Source: Beilstein J Nanotechnol. 2012 Feb 20;3:144–62. doi: 10.3762/bjnano.3.15 (PMC3304326; doi:10.3762/bjnano.3.15)
Supplement: File 1 — Useful mathematical relations and detailed calculations [file Beilstein_J_Nanotechnol-03-144-s001.pdf]

Supporting Information

for

Current-induced forces in mesoscopic systems: A scattering matrix approach

Niels Bode[1], Silvia Viola Kusminskiy*[1], Reinhold Egger[2] and Felix von Oppen[1]

Address: [1]Dahlem Center for Complex Quantum Systems and Fachbereich Physik, Freie Universität Berlin, 14195 Berlin, Germany and [2]Institut für Theoretische Physik, Heinrich-Heine-Universität, D-40225 Düsseldorf, Germany

Email: Silvia Viola Kusminskiy* - silvia.kusminskiy@fu-berlin.de

∗ Corresponding author

Useful mathematical relations and detailed calculations

A Useful relations

Here we list a set of useful relations for the derivations in the main text.

A.1 Green's functions relations

The Green's functions are related via

$$G^R - G^A = G^> - G^<. \tag{87}$$

The lesser and larger Green's functions are given by

$$G^< = G^R \Sigma^< G^A = 2i \sum_\alpha f_\alpha \, G^R \Gamma_\alpha G^A = 2\pi i \sum_\alpha f_\alpha \, G^R W^\dagger \Pi_\alpha W G^A \tag{88}$$

$$G^> = G^< + G^R - G^A = -2\pi i \sum_\alpha (1 - f_\alpha) \, G^R W^\dagger \Pi_\alpha W G^A. \tag{89}$$

From Equation 22 it is easy to see that

$$W^\dagger W = \frac{1}{2\pi i}[(G^R)^{-1} - (G^A)^{-1}], \tag{90}$$

$$\partial_{X_\nu} G^R = G^R \Lambda_\nu G^R \tag{91}$$

and

$$\partial_\varepsilon G^R = -G^R (1 - \partial_\varepsilon \Sigma^R) G^R. \tag{92}$$

A.2 Green's functions and S-matrix relations

Noting that (for given t) $\partial_{X_\nu} G^R = G^R \Lambda_\nu G^R$, we find using Equation 90:

$$S^\dagger \frac{\partial S}{\partial X_\nu} = -2\pi i (1 + 2\pi i W G^A W^\dagger) W G^R \Lambda_\nu G^R W^\dagger = -2\pi i W G^A \Lambda_\nu G^R W^\dagger. \tag{93}$$

This holds for arbitrary magnitude of X_ν.

In the main text we use

$$\frac{1}{\pi}\frac{\partial S^\dagger}{\partial X_\nu}A_{\nu'} = 2\pi i W G^A \Lambda_\nu G^A W^\dagger \partial_\varepsilon (W G^R)\Lambda_{\nu'} G^R W^\dagger - W G^A \Lambda_\nu (G^A - G^R)\Lambda_{\nu'}\partial_\varepsilon (G^R W^\dagger), \tag{94}$$

$$\pi\left(\left[S^\dagger \frac{\partial S}{\partial X_\nu}, W G^A \Lambda_{\nu'} \frac{\partial (G^R W^\dagger)}{\partial_\varepsilon} - \frac{\partial (W G^A)}{\partial_\varepsilon}\Lambda_{\nu'} G^R W^\dagger\right]_-\right)_s = \left(\frac{\partial S^\dagger}{\partial X_\nu}A_{\nu'} - A^\dagger_{\nu'}\frac{\partial S}{\partial X_\nu}\right)_s \tag{95}$$

and

$$\left[S^\dagger \frac{\partial A_\nu}{\partial X_{\nu'}}\right]_a = -2\pi\left[W G^A \Lambda_\nu (\partial_\varepsilon G^R)\Lambda_{\nu'} G^R W^\dagger\right]_a . \tag{96}$$

For energy-independent Γ^α, we can use Equation 92 so that also

$$S^\dagger \frac{\partial S}{\partial \varepsilon} = 2\pi i W G^A G^R W^\dagger, \tag{97}$$

$$\partial_\varepsilon \left(S^\dagger \frac{\partial S}{\partial X_\nu}\right) = 2\pi i W G^A \left(G^A \Lambda_\nu + \Lambda_\nu G^R\right) G^R W^\dagger \tag{98}$$

and Equation 94 simplifies to

$$\frac{\partial S^\dagger}{\partial X_\nu}A_{\nu'} = \pi W G^A \Lambda_\nu \left(G^A - G^R\right)\left(\Lambda_{\nu'} G^R - G^R \Lambda_{\nu'}\right) G^R W^\dagger . \tag{99}$$

B S-matrix derivation of the damping matrix

The expression for γ^s given in Equation 27 can be written explicitly in terms of retarded and advanced Green's functions as

$$\gamma^s_{\nu\nu'} = 2\pi \sum_{\alpha\alpha'} \int d\varepsilon f_\alpha \mathrm{tr}\left\{\Lambda_\nu G^R W^\dagger \Pi_\alpha W G^A \Lambda_{\nu'} \partial_\varepsilon \left[(1 - f_{\alpha'}) G^R W^\dagger \Pi_{\alpha'} W G^A\right]\right\}_s . \tag{100}$$

We split Equation 100 into two terms, the first due to the derivative acting on the Fermi function, the second from the rest, $\gamma^s = \gamma^{s(I)} + \gamma^{s(II)}$. The first term is given by

$$\gamma^{s(I)}_{\nu\nu'} = 2\pi \sum_{\alpha\alpha'} \int d\varepsilon f_\alpha (-\partial_\varepsilon f_{\alpha'}) \mathrm{tr}\left\{\Pi_{\alpha'} W G^A \Lambda_\nu G^R W^\dagger \Pi_\alpha W G^A \Lambda_{\nu'} G^R W^\dagger\right\}_s \tag{101}$$

where we have used the cyclic invariance of the trace. Similar to the derivation for the mean force, by means of Equation 93, Equation 101 can be expressed in terms of the frozen S-matrix as

$$\gamma^{s(I)}_{\nu\nu'} = -\sum_{\alpha\alpha'} \int \frac{\mathrm{d}\varepsilon}{2\pi} f_\alpha (-\partial_\varepsilon f_{\alpha'}) \mathrm{Tr}\left\{\Pi_\alpha S^\dagger \frac{\partial S}{\partial X_\nu} \Pi_{\alpha'} S^\dagger \frac{\partial S}{\partial X_{\nu'}}\right\}_s . \tag{102}$$

The second contribution, in terms of G^R and G^A, reads

$$\gamma^{s(II)}_{\nu\nu'} = (2\pi)^2 \sum_{\alpha\alpha'} \int \frac{\mathrm{d}\varepsilon}{2\pi} F_{\alpha\alpha'} \mathrm{tr}\left\{\Lambda_\nu G^R W^\dagger \Pi_\alpha W G^A \Lambda_{\nu'} \partial_\varepsilon \left(G^R W^\dagger \Pi_{\alpha'} W G^A\right)\right\}_s . \tag{103}$$

It is instructive to split the factor $F_{\alpha\alpha'}$ into a symmetric and an antisymmetric part under exchange of the lead indices, $F_{\alpha\alpha'} = F^s_{\alpha\alpha'} + F^a_{\alpha\alpha'}$, with

$$\begin{aligned} F^s_{\alpha\alpha'} &\equiv \frac{1}{2}(f_\alpha + f_{\alpha'} - 2 f_\alpha f_{\alpha'}) \\ F^a_{\alpha\alpha'} &\equiv \frac{1}{2}(f_\alpha - f_{\alpha'}) . \end{aligned} \tag{104}$$

Correspondingly, we split $\gamma^{s(II)}$ into symmetric $\left[\gamma^{s(IIs)}\right]$ and antisymmetric $\left[\gamma^{s(IIa)}\right]$ parts in the lead indices: $\gamma^{s(II)} = \gamma^{s(IIs)} + \gamma^{s(IIa)}$. Due to its symmetries, $\gamma^{s(IIs)}$ can be easily expressed in terms of the S-matrix,

$$\begin{aligned} \gamma^{s(IIs)}_{\nu\nu'} &= \pi \sum_{\alpha\alpha'} \int d\varepsilon F^s_{\alpha\alpha'} \partial_\varepsilon \mathrm{tr}\left\{\Lambda_\nu G^R W^\dagger \Pi_\alpha W G^A \Lambda_{\nu'} G^R W^\dagger \Pi_{\alpha'} W G^A\right\}_s \\ &= -\pi \sum_{\alpha\alpha'} \int d\varepsilon \, (\partial_\varepsilon F^s_{\alpha\alpha'}) \, \mathrm{tr}\left\{\Lambda_\nu G^R W^\dagger \Pi_\alpha W G^A \Lambda_{\nu'} G^R W^\dagger \Pi_{\alpha'} W G^A\right\}_s \\ &= \frac{1}{4\pi} \sum_{\alpha\alpha'} \int d\varepsilon \, (\partial_\varepsilon F^s_{\alpha\alpha'}) \, \mathrm{tr}\left\{\Pi_\alpha S^\dagger \frac{\partial S}{\partial X_\nu} \Pi_{\alpha'} S^\dagger \frac{\partial S}{\partial X_{\nu'}}\right\}_s \end{aligned} \tag{105}$$

where in the second line we have integrated by parts since F^s vanishes for $\varepsilon \to \pm\infty$, and in the last line we have used Equation 93 once again.

B.1 "Equilibrium" dissipative term $\gamma^{s,eq}$

Since in equilibrium $F^a_{\alpha\alpha'} = F^a_{\alpha\alpha} = 0$, $\gamma^{s(IIa)}\Big|_{eq} = 0$ and we can now regroup terms into an "equilibrium" contribution, $\gamma^{s,eq} = \gamma^{s(I)} + \gamma^{s(IIs)}$, and a purely non-equilibrium contribution $\gamma^{s,ne} \equiv \gamma^{s(IIa)}$:

$$\gamma^s = \gamma^{s,eq} + \gamma^{s,ne}\,. \tag{106}$$

By adding up the expressions in Equation 102 and Equation 105, it is straightforward to obtain Equation 44 for $\gamma^{s,eq}$ given in the main text.

B.2 Non-equilibrium dissipative term $\gamma^{s,ne}$

To obtain $\gamma^{s,ne}$ in terms of S-matrix quantities we start from the expression

$$\gamma^{s,ne}_{\nu\nu'} = 2\pi \sum_{\alpha\alpha'} \int \mathrm{d}\varepsilon F^a_{\alpha\alpha'} \mathrm{tr}\left\{\Lambda_\nu G^R W^\dagger \Pi_\alpha W G^A \Lambda_{\nu'} \partial_\varepsilon \left(G^R W^\dagger \Pi_{\alpha'} W G^A\right)\right\}_s, \tag{107}$$

and exploiting $\sum_\alpha \Pi_\alpha = 1$ and the identity (Equation 93), we note that Equation 107 can be written as

$$\gamma^{s,ne}_{\nu\nu'} = -\frac{i}{2} \int \mathrm{d}\varepsilon \sum_\alpha f_\alpha \mathrm{tr}\left\{\Pi_\alpha \left[S^\dagger \frac{\partial S}{\partial X_\nu}, W G^A \Lambda_{\nu'} \frac{\partial (G^R W^\dagger)}{\partial \varepsilon} - \frac{\partial (W G^A)}{\partial \varepsilon} \Lambda_{\nu'} G^R W^\dagger\right]\right\}_s, \tag{108}$$

where $[.,.]$ indicates the commutator. Calculating each term in the commutator separately we obtain

$$\begin{aligned}
S^\dagger \frac{\partial S}{\partial X_\nu}\left[WG^A\Lambda_{\nu'}\frac{\partial (G^R W^\dagger)}{\partial \varepsilon}-\frac{\partial (WG^A)}{\partial \varepsilon}\Lambda_{\nu'}G^R W^\dagger\right] &= -WG^A\Lambda_\nu (G^A-G^R)\Lambda_{\nu'}\frac{\partial (G^R W^\dagger)}{\partial \varepsilon}\\
&+2\pi i WG^A\Lambda_\nu G^R W^\dagger \frac{\partial (WG^A)}{\partial \varepsilon}\Lambda_{\nu'}G^R W^\dagger\\
\left[WG^A\Lambda_{\nu'}\frac{\partial (G^R W^\dagger)}{\partial \varepsilon}-\frac{\partial (WG^A)}{\partial \varepsilon}\Lambda_{\nu'}G^R W^\dagger\right] S^\dagger \frac{\partial S}{\partial X_\nu} &= -\frac{\partial (WG^A)}{\partial \varepsilon}\Lambda_{\nu'}(G^A-G^R)\Lambda_\nu G^R W^\dagger\\
&-2\pi i WG^A\Lambda_{\nu'}\frac{\partial (G^R W^\dagger)}{\partial \varepsilon} WG^A\Lambda_\nu G^R W^\dagger ,
\end{aligned} \tag{109}$$

where we have used Equation 90. Finally, with help of the identity (Equation 94), the non-equilibrium term can be expressed as Equation 45 in the main text.

C Resonant level forces: alternative expressions

To calculate the current-induced forces for the resonant level model presented in Section "Applications", we can alternatively start with the popular S-matrix parametrization [1,2]

$$S=\begin{pmatrix} \sqrt{1-\mathscr{T}}e^{i\theta} & \sqrt{\mathscr{T}}e^{i\eta} \\ \sqrt{\mathscr{T}}e^{i\eta} & -\sqrt{1-\mathscr{T}}e^{i(2\eta-\theta)} \end{pmatrix}, \tag{110}$$

where the transmission coefficient $\mathscr{T}$ and the phases η,θ depend on X. We present here the results for linear coupling, $\tilde{\varepsilon}(X)=\varepsilon_0+\lambda X$. We can then identify the transmission probability

$$\mathscr{T}(\varepsilon,X)=\frac{4\Gamma_L\Gamma_R}{(\varepsilon-\varepsilon_0-\lambda X)^2+\Gamma^2} \tag{111}$$

and the phases

$$\begin{aligned}
\eta(\varepsilon,X) &= -\frac{\pi}{2}-\arctan\left(\frac{\Gamma}{\varepsilon-\varepsilon_0-\lambda X}\right)\\
\theta(\varepsilon,X) &= \frac{\pi}{2}+\eta+\arctan\left(\frac{\Gamma_R-\Gamma_L}{\varepsilon-\varepsilon_0-\lambda X}\right).
\end{aligned}$$

We can now relate the current-induced forces to this S-matrix parametrization. The result for the average force can be split into a non-equilibrium force F^{ne} and an equilibrium force F^{eq}, *i.e.*, $F = F^{ne} + F^{eq}$ with

$$\begin{aligned} F^{ne}(X) &= \int \frac{d\varepsilon}{2\pi}(f_L - f_R)(1-\mathscr{T})\frac{\partial(\theta-\eta)}{\partial X} \\ F^{eq}(X) &= \int \frac{d\varepsilon}{2\pi}(f_L + f_R)\frac{\partial \eta}{\partial X}\,. \end{aligned} \tag{112}$$

The amplitude of the fluctuating force can be obtained from Equation 42 and is given by

$$D(X) = \int \frac{d\varepsilon}{2\pi} \sum_{\alpha\alpha'} F^s_{\alpha\alpha'} Y_{\alpha\alpha'}\,, \tag{113}$$

where we have defined

$$\begin{aligned} Y_{LL} &= \left[(1-\mathscr{T})\frac{\partial(\eta-\theta)}{\partial X} - \frac{\partial \eta}{\partial X}\right]^2 \\ Y_{RR} &= \left[(1-\mathscr{T})\frac{\partial(\eta-\theta)}{\partial X} + \frac{\partial \eta}{\partial X}\right]^2 \\ Y_{LR} &= Y_{RL} = \frac{1}{4\mathscr{T}(1-\mathscr{T})}\left(\frac{\partial \mathscr{T}}{\partial X}\right)^2 + \mathscr{T}(1-\mathscr{T})\left(\frac{\partial(\eta-\theta)}{\partial X}\right)^2. \end{aligned}$$

After some algebra, we also obtain

$$\gamma^s(X) = \frac{1}{2T}\left[D(X) - \int \frac{d\varepsilon}{2\pi}(f_L - f_R)^2 Y_{LR}\right]. \tag{114}$$

This last expression corresponds to $\gamma^{s,eq}$ given in Equation 44. (As we pointed out previously, $\gamma^{s,ne}$ vanishes in this case). Here we have isolated a term that vanishes in equilibrium, showing explicitly that there is a non-equilibrium contribution in 44.

D Current-induced forces for the two-level model

The mean force is given by

$$F(X) = -\lambda_1 \Gamma \int \frac{d\varepsilon}{2\pi} \left[(f_L + f_R) \frac{2\lambda_1 X(\varepsilon - \varepsilon_0)}{|\Delta|^2} + (f_L - f_R) \frac{(\varepsilon - \varepsilon_0)^2 + (\lambda_1 X)^2 - t^2 + (\Gamma/2)^2}{|\Delta|^2} \right] . \tag{115}$$

The friction coefficient $\gamma^s = \gamma^{s,eq} + \gamma^{s,ne}$ reads

$$\begin{aligned}
\gamma^{s,eq} =& \frac{\lambda_1^2 \Gamma^2}{4\pi} \int d\varepsilon \left\{ -\frac{\partial_\varepsilon f_L + \partial_\varepsilon f_R}{|\Delta|^4} \Big[\left((\varepsilon - \varepsilon_0)^2 + (\Gamma/2)^2 + (\lambda_1 X)^2 + t^2 \right)^2 + (2(\varepsilon - \varepsilon_0)\lambda_1 X)^2 \right. \\
& \left. - (2(\varepsilon - \varepsilon_0)t)^2 \Big] + \frac{\partial_\varepsilon f_R - \partial_\varepsilon f_L}{|\Delta|^4} \left[4(\varepsilon - \varepsilon_0)\lambda_1 X \left((\varepsilon - \varepsilon_0)^2 + (\Gamma/2)^2 + (\lambda_1 X)^2 - t^2 \right) \right] \right\} , \\
\gamma^{s,ne} =& \frac{2\lambda_1^2 \Gamma^2 t^2 \lambda_1 X}{\pi} \int d\varepsilon \frac{f_R - f_L}{|\Delta|^6} \Big[\left((\varepsilon - \varepsilon_0)^2 - (\lambda_1 X)^2 - t^2 \right)^2 \\
& + 2(\Gamma/2)^2 \left((\varepsilon - \varepsilon_0)^2 + (\lambda_1 X)^2 + t^2 \right) + (\Gamma/2)^4 \Big] .
\end{aligned} \tag{116}$$

E Current-induced forces for the two vibrational modes model

Here we list the current-induced forces quantities, calculated from Equations 39, 42, 47 and 50 for the two-modes example discussed in the main text. For convenience, we define the following quantities:

$$g_{\alpha 0}(\varepsilon) = \frac{(\varepsilon - \tilde{\varepsilon})^2 + \tilde{t}^2 + \Gamma_{1-\alpha}^2}{|\Delta|^2} \tag{117}$$

$$g_{\alpha 1}(\varepsilon) = \frac{2\tilde{t}(\varepsilon - \tilde{\varepsilon})}{|\Delta|^2} \tag{118}$$

$$g_{\alpha 2}(\varepsilon) = \pm \frac{-2\tilde{t}\Gamma_{1-\alpha}}{|\Delta|^2} \tag{119}$$

$$g_{\alpha 3}(\varepsilon) = \pm \frac{(\varepsilon - \tilde{\varepsilon})^2 + \Gamma_{1-\alpha}^2 - \tilde{t}^2}{|\Delta|^2} \tag{120}$$

where the $+(-)$ refers to $\alpha = L(R)$ and with $1-\alpha = R(L)$ for $\alpha = L(R)$, and $\Delta(X_1,X_2) = (\varepsilon - \tilde{\varepsilon} + i\Gamma_L)(\varepsilon - \tilde{\varepsilon} + i\Gamma_R) - \tilde{t}^2$.

E.1 Mean force

$$F_1 = -2\int \frac{d\varepsilon}{2\pi}\lambda_1 \sum_\alpha \frac{f_\alpha(\varepsilon)\Gamma_\alpha\left((\varepsilon-\tilde{\varepsilon})^2+\tilde{t}^2+\Gamma_{1-\alpha}^2\right)}{\left[(\varepsilon-\tilde{\varepsilon})^2-\tilde{t}^2-\Gamma_L\Gamma_R\right]^2+\left[(\Gamma_L+\Gamma_R)(\varepsilon-\tilde{\varepsilon})\right]^2} \quad (121)$$

$$F_2 = -4\int \frac{d\varepsilon}{2\pi}\lambda_2 \frac{\tilde{t}(\varepsilon-\tilde{\varepsilon})\left(f_L(\varepsilon)\Gamma_L+f_R(\varepsilon)\Gamma_R\right)}{\left[(\varepsilon-\tilde{\varepsilon})^2-\tilde{t}^2-\Gamma_L\Gamma_R\right]^2+\left[(\Gamma_L+\Gamma_R)(\varepsilon-\tilde{\varepsilon})\right]^2} \quad (122)$$

E.2 Fluctuating force

$$D_{11} = 2(\lambda_1)^2\int \frac{d\varepsilon}{2\pi}\sum_{\alpha\beta} f_\alpha(\varepsilon)\Gamma_\alpha\left(1-f_\beta(\varepsilon)\right)\Gamma_\beta\sum_\mu g_{\alpha\mu}g_{\beta\mu} \quad (123)$$

$$D_{12} = 2\lambda_1\lambda_2\int \frac{d\varepsilon}{2\pi}\sum_{\alpha\beta} f_\alpha(\varepsilon)\Gamma_\alpha\left(1-f_\beta(\varepsilon)\right)\Gamma_\beta\left(g_{\alpha0}g_{\beta1}+g_{\alpha1}g_{\beta0}\right) \quad (124)$$

$$D_{22} = 2(\lambda_2)^2\int \frac{d\varepsilon}{2\pi}\sum_{\alpha\beta} f_\alpha(\varepsilon)\Gamma_\alpha\left(1-f_\beta(\varepsilon)\right)\Gamma_\beta\left(g_{\alpha0}g_{\beta0}+g_{\alpha1}g_{\beta1}-g_{\alpha2}g_{\beta2}-g_{\alpha3}g_{\beta3}\right) \quad (125)$$

E.3 Damping coefficients

$$\gamma_{11}^s = \frac{(\lambda_1)^2}{2\pi}\int d\varepsilon\sum_{\alpha\beta}\left(-\partial_\varepsilon f_\alpha(\varepsilon)\right)\Gamma_\alpha\Gamma_\beta\sum_\mu g_{\alpha\mu}g_{\beta\mu} \quad (126)$$

$$\gamma_{12}^s = 2\lambda_1\lambda_2\int \frac{d\varepsilon}{2\pi}\sum_{\alpha\beta} f_\alpha(\varepsilon)\Gamma_\alpha\left(-\partial_\varepsilon f_\beta(\varepsilon)\right)\Gamma_\beta\left(g_{\alpha0}g_{\beta1}+g_{\alpha1}g_{\beta0}\right) \quad (127)$$

$$\gamma_{22}^s = 2(\lambda_2)^2\int \frac{d\varepsilon}{2\pi}\sum_{\alpha\beta} f_\alpha(\varepsilon)\Gamma_\alpha\left(-\partial_\varepsilon f_\beta(\varepsilon)\right)\Gamma_\beta\left(g_{\alpha0}g_{\beta0}+g_{\alpha1}g_{\beta1}-g_{\alpha2}g_{\beta2}-g_{\alpha3}g_{\beta3}\right) \quad (128)$$

E.4 "Lorentz" term

$$\gamma_{12}^a = -2\tilde{t}\frac{\lambda_1\lambda_2}{\pi}\Gamma_L\Gamma_R(\Gamma_L^2-\Gamma_R^2)\int d\varepsilon\left[\partial_\varepsilon\frac{\varepsilon-\tilde{\varepsilon}}{|\Delta|^2}\right]\left[\frac{f_L-f_R}{|\Delta|^2}\right] \quad (129)$$

References

1. Nazarov, Y.; Blanter, Y. *Quantum Transport*; Cambridge University Press: Cambridge, UK, 2010.

2. Bennett, S. D.; Maassen, J.; Clerk, A. A. *Phys. Rev. Lett.* **2010**, *105*, 217206. doi:10.1103/PhysRevLett.105.217206 (See also *Phys. Rev. Lett.* **2011,** *106,* 199902.)
